# Population level multimodal neuroimaging correlates of attention-deficit hyperactivity disorder among children

**DOI:** 10.3389/fnins.2023.1138670

**Published:** 2023-02-22

**Authors:** Huang Lin, Stefan P. Haider, Simone Kaltenhauser, Ali Mozayan, Ajay Malhotra, R. Todd Constable, Dustin Scheinost, Laura R. Ment, Kerstin Konrad, Seyedmehdi Payabvash

**Affiliations:** ^1^Department of Radiology and Biomedical Imaging, Yale School of Medicine, New Haven, CT, United States; ^2^Child Neuropsychology Section, Department of Child and Adolescent Psychiatry, Psychosomatics and Psychotherapy, University Hospital RWTH Aachen, Aachen, Germany; ^3^Department of Pediatrics, Yale School of Medicine, New Haven, CT, United States; ^4^Department of Neurology, Yale School of Medicine, New Haven, CT, United States; ^5^Jülich Research Centre, JARA Brain Institute II, Molecular Neuroscience and Neuroimaging (INM-11), Jülich, Germany

**Keywords:** attention-deficient hyperactivity disorder, brain connectivity, white matter microstructure, cortex morphology, machine learning

## Abstract

**Objectives:**

Leveraging a large population-level morphologic, microstructural, and functional neuroimaging dataset, we aimed to elucidate the underlying neurobiology of attention-deficit hyperactivity disorder (ADHD) in children. In addition, we evaluated the applicability of machine learning classifiers to predict ADHD diagnosis based on imaging and clinical information.

**Methods:**

From the Adolescents Behavior Cognitive Development (ABCD) database, we included 1,798 children with ADHD diagnosis and 6,007 without ADHD. In multivariate logistic regression adjusted for age and sex, we examined the association of ADHD with different neuroimaging metrics. The neuroimaging metrics included fractional anisotropy (FA), neurite density (ND), mean-(MD), radial-(RD), and axial diffusivity (AD) of white matter (WM) tracts, cortical region thickness and surface areas from T1-MPRAGE series, and functional network connectivity correlations from resting-state fMRI.

**Results:**

Children with ADHD showed markers of pervasive reduced microstructural integrity in white matter (WM) with diminished neural density and fiber-tracks volumes – most notable in the frontal and parietal lobes. In addition, ADHD diagnosis was associated with reduced cortical volume and surface area, especially in the temporal and frontal regions. In functional MRI studies, ADHD children had reduced connectivity among default-mode network and the central and dorsal attention networks, which are implicated in concentration and attention function. The best performing combination of feature selection and machine learning classifier could achieve a receiver operating characteristics area under curve of 0.613 (95% confidence interval = 0.580–0.645) to predict ADHD diagnosis in independent validation, using a combination of multimodal imaging metrics and clinical variables.

**Conclusion:**

Our study highlights the neurobiological implication of frontal lobe cortex and associate WM tracts in pathogenesis of childhood ADHD. We also demonstrated possible potentials and limitations of machine learning models to assist with ADHD diagnosis in a general population cohort based on multimodal neuroimaging metrics.

## 1. Introduction

Attention-deficit/hyperactivity disorder (ADHD) is estimated to affect 6.1% of children in the U.S. (Danielson et al., 2018). Neuroimaging studies can help elucidate the underlying neurobiology of ADHD, suggesting that abnormal brain connectivity plays a central role in pathogenesis of ADHD (Aoki et al., 2018). Children with ADHD also have shown abnormalities in axonal density and volume of multiple white matter (WM) tracts (Wu et al., 2020). In addition, subtle differences in cortical surface area, involvement of the frontal cortex and reduced cortical volume (Kumar et al., 2017) as well as alterations in functional connectivity in the left insula and left inferior frontal gyrus (Chiang et al., 2020) have been reported in children with ADHD. The variable range of reported neuroimaging correlates of ADHD may be due to small sample size and differences in diagnostic criteria of prior studies. Large studies – such as the ABCD (Adolescent Brain and Cognitive Development) – can provide powerful tools to determine neuroimaging correlates of ADHD among the general population children.

In this study, we aimed to determine the imaging metrics of brain microstructure, morphology and functional connectivity associated with ADHD diagnosis in a large cross-sectional cohort of preadolescent American children. While prior large-scale studies focused on one set of neuroimaging characteristics (e.g., cortical thickness) in relation to ADHD diagnosis (Hoogman et al., 2019; Bernanke et al., 2022; Sudre et al., 2022), we examined multimodal imaging metrics among the same children cohort to achieve a comprehensive assessment of brain morphology, microstructure and connectivity changes in associated with ADHD. We also trained, finetuned, compared, and validated different combinations of feature selection and machine learning classifiers to predict ADHD diagnosis in children based on multimodal MRI metrics. Such neuroimaging-based tools may complement the clinical assessment for the diagnosis of ADHD among children, particularly in the presence of cultural, language, or communication barriers.

## 2. Materials and methods

### 2.1. The ABCD database and study population

The ABCD Study (RRID: SCR_015769) is the largest longitudinal study of neurodevelopment and child health in the United States. Using a school-based recruitment strategy, data from over ten thousand 9–10-year-olds were collected from 21 sites, which included multimodal neuroimaging, and standardized cognitive and clinical assessments (Casey et al., 2018). The study population is representative of the demographics of the general U.S. population (Garavan et al., 2018). The inclusion criteria were children’s age and attending a public or private elementary school in the catchment area; whereas, exclusion criteria were: (1) child not being proficient in English language; (2) having severe limitations in sensory, neurological, medical, or intellectual abilities that would prevent the child from following the study protocol; and (3) not being able to complete a baseline MRI scan. The study adheres to the policies and procedures of the Institutional Review Board at each site, and all participants have given their informed consent (for parents) or assent (for children) to participate.

### 2.2. Subjects’ ascertainment

**FIGURE 1:**
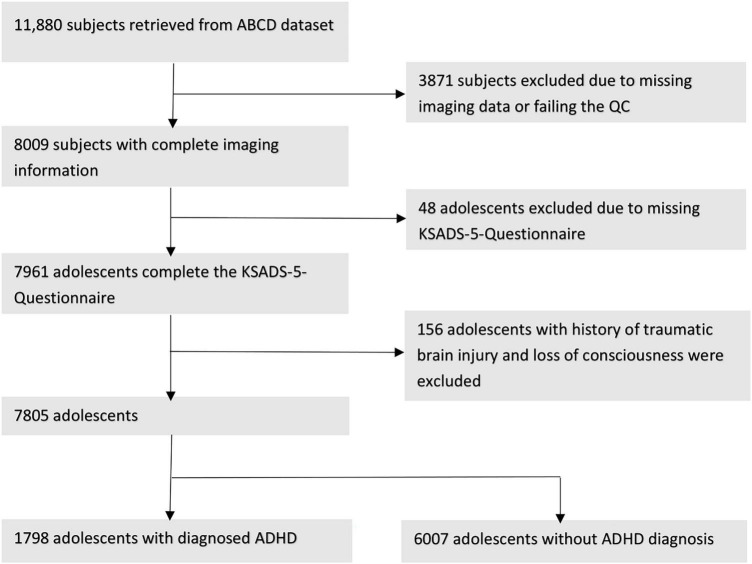
Workflow after we proceed to extract our cohorts regarding to the exclusion criteria and showing the remain subjects.

Figure 1 summarizes the subjects’ ascertainment process. We retrieved the tabulated imaging information and clinical information from the third public ABCD data release (Yang and Jernigan, 2020) including baseline and the 2-year follow-up assessments. Following the recommendations of the ABCD Consortium in the Release Notes to the 3.0 release, we removed all patients who had an fMRI scan using Philips scanners due to incorrect post-processing (Yang and Jernigan, 2020). To identify patients with ADHD, we used the ABCD Parent Diagnostic Interview scale for the Kiddie-Schedule for Affective Disorders and Schizophrenia (K-SADS) DSM-5 to label the children as having ADHD or not. For assessment, the computerized version of the KSADS (KSADS-COMP) was used by the ABCD Consortium. We selected the KSADS-COMP categorical diagnosis of ADHD as the primary outcome due to its availability in the ABCD dataset, alignment with DSM-5 criteria, established validity and reliability in both research and clinical settings, including in epidemiological studies (Kaufman et al., 1997). Anyone with a present, past or (partial) remission ADHD diagnosis at baseline or 2-year follow-up assessment was assigned to the ADHD-positive group, and remaining subjects were labeled as “without ADHD.” We included following covariates from the ABCD dataset: children’s age (months), biological sex (female, male), race (White, Black, Asian, Hispanic, Mixed/Other), highest parental education (no Highschool Degree, Highschool/General Education Degree, College/Associate Degree, Bachelor’s Degree, Postgraduate Degree), and handedness (Right, Left, Mixed). Biological sex, race and highest parental education were assessed by parental reported questionnaires at baseline. Handedness was assessed at baseline using the Youth Edinburgh Handedness Inventory Short Form. We excluded those with incomplete K-SADS ADHD diagnosis at baseline or 2-year-follow-up, history of traumatic brain injury, and failure to pass the image quality control performed manually by the ABCD Consortium (e.g., due to artifacts) (Hagler et al., 2019).

### 2.3. MRI acquisition and neuroimaging measures

The ABCD image acquisition protocol includes structural (sMRI), diffusion (dMRI), and functional (fMRI) MRI collected using Siemens Prisma, GE 750 and Philips Achieva and Ingenia 3 T scanners. According to the ABCD Consortium, standardized imaging protocols were used to create harmonized scans across all sites and scanner manufactures.^1^ Full details on image acquisition and image processing steps can be found in paper provided by the ABCD Consortium (Casey et al., 2018; Hagler et al., 2019). Details about acquisition parameter have been described previously (Casey et al., 2018). The standardized processing pipeline was performed with Freesurfer (version 5.3.0). A list of all cortical, subcortical, and WM regions of interest (ROI) can be found in section “1. List of Atlas Regions (Supplementary material).” In the following section we will give a brief overview.

#### 2.3.1. Structural MRI

Morphometric and macrostructural properties of different cortical regions of interest (ROI) based on the Desikan-Killiany atlas (Desikan et al., 2006) were extracted from high resolution T1-weighted images (1 mm isotropic) – including cortical volume, thickness, surface areas, and sulcal depths of 35 ROI. The standardized processing included skull stripping, WM segmentation, topological defect correction, surface optimization, and non-linear registration to a spherical surface-based atlas.

#### 2.3.2. Diffusion MRI

The dMRI data was collected in the axial plane using a resolution of 1.7 mm in all directions, which is the same as the dMRI data acquisition resolution, and a multiband acceleration factor of 3. Images were corrected for eddy current distortion, head motion, spatial, and intensity distortion correction and gradient non-linearity correction. The dMRI data was aligned with T1w structural images using mutual information after a rough initial alignment with atlas brains. To examine WM microstructural integrity, neurite density (ND) and fiber tract (FT) thickness as well as water diffusion metrics – including fractional anisotropy (FA), mean- (MD), longitudinal- (RD), and axial diffusivity (AD) – were calculated for 35 WM tracts and only ND for 12 subcortical structures from dMRI scans. These metrics from major WM tracts were extracted using AtlasTrack implemented (Hagler et al., 2009).

#### 2.3.3. Resting-state fMRI

High resolution resting state fMRI data was acquired in four runs of 5 min each, for a total of 20 min. Other resting-state parameters have been previously described.^2^ In the ABCD study, a software package called Multi-Modal Processing Stream (MMPS) is used for pre-processing data and incorporates other software packages such as FreeSurfer, AFNI, and FSL. The pre-processing steps include correcting head motion by aligning each frame with the first one, fixing B0 distortions, and resampling the scans to align with each other. The scans are also registered to T1-weighted structural images using mutual information. Linear regression is used to remove quadratic trends, signals that are correlated with estimated motion time courses, and average time courses for WM, ventricles, and the whole brain. Motion regression includes six parameters and their derivatives and squares, and only frames with framewise displacement (FD) less than 0.3 mm are included. Time points with FD more than 0.2 mm are excluded from variance and correlation calculations. The average FD is 0.25 mm. The time courses are filtered between 0.009 and 0.08 Hz. Motion censoring is applied to reduce remaining effects of head motion that survive the pre-analysis regression. Additional censoring is applied based on detecting time points as outliers with respect to spatial variation across the brain to account for lingering effects of head motion. The average correlation between the baseline functional networks according to Gordon parcel atlas (Gordon et al., 2016), were calculated and transformed to *z*-scores, representing the functional connectivity.

### 2.4. Multivariate models

In separate multivariate logistic regression models, we tested the association of different neuroimaging metrics with presence versus absence of ADHD. In each model, we tested the association of one neuroimaging variable with ADHD presence after correction for children’s age, biological sex, race, highest parental education and handedness as covariates. Biological sex, race and highest parental education were assessed by parental reported questionnaires. Handedness was assessed by the Youth Edinburgh Handedness Inventory Short Form. All variables were treated as unordered factors, except of age. The neuroimaging metrics included the averaged FA, ND, MD, RD, FT, and AD of 35 WM tracts and ND of 12 subcortical regions, thickness, and surface areas of 68 cortical regions, the inter- and intra-network correlations of 13 functional networks. To correct for multiple comparisons, we applied false discovery rate separately for each neuroimaging metrics to generate adjusted *p*-values. *p*-Values < 0.05 were considered as significant. All analyses were performed using R software (version 4.1.2) using the *stats*-package (version 3.6.2).

### 2.5. Feature selection and machine learning

We trained, finetuned, and compared combinations of six different machine learning classifiers and five feature selection methods to predict the presence versus absence of ADHD. The input included all multimodal MRI metrics with and without the clinical information (i.e., sex, age, race, highest parental education, and handedness). Detailed description of the different machine learning algorithms and featured selection methods are in section “2. Machine learning (Supplementary material)” and reported previously (Haider et al., 2020). The dataset was randomly split into training/cross-validation and independent validation cohorts in a 4-to-1 ratio. Selection of features were done on the training folds before training the machine-learning classifier to prevent information leakage and reduce overfitting. In every iteration of the cross-validation process, the training data was standardized and certain features were selected, and then a machine learning model was trained using that data. This method ensures accurate estimates of the model’s performance on independent validation sets. For each combination we created a framework of five repeats of 10-fold cross-validation for finetuning of feature selection and machine learning hyperparameters using the Bayesian optimization within the training/cross-validation cohort. For every hyperparameter and the number of selected features, an upper and lower bond was set as described previously (see section “2.3. Hyperparameter bounds for Bayesian optimization (Supplementary material)”; Haider et al., 2020). The optimized parameters were then adopted in the machine-learning/feature-selection framework followed by five repeats of 10-fold cross-validation. In each fold of cross-validation, we calculated the area under curve (AUC) of receiver operating characteristics (ROC) for prediction of ADHD presence in validation fold. The averaged AUC across all validation folds was used to identify the best combination. Finally, we trained the best performing model with optimized parameters on all training/cross-validation cohort, and tested the model in independent validation set, which was isolated from the training, optimization, and cross-validation process. The trained model was applied to the independent test set and the performance was evaluate by AUC (95% CI).

## 3. Results

### 3.1. Subjects’ demographics

Figure 1 depicts the inclusion flowchart of children for our analysis. After excluding those with incomplete clinical information, history of traumatic brain injury, and failure to pass image quality control, a total of 7,805 participants (average age of 119.33 months) were included in our analysis – 1,798 with and 6,007 without ADHD. Table 1 summarizes demographic characteristics of each subcohort. In multivariate analysis, there was a significant difference in racial/ethnicity distribution between those with and without ADHD (Table 1), with ratio of black children slightly higher while Asian children lower among those with ADHD. In addition, the rate of handedness and parental education were different between children with and without ADHD (Table 1).

**TABLE 1:** Characteristics of children with and without ADHD diagnosis.

Demographics description				
	With ADHD	Without ADHD	Total	*p*-value
	1,798	6,007	7,805	
Age (in months)				0.83
Mean (SD)	119.5 (7.5)	119.3 (7.6)	119.3 (7.55)	
Min–max	107–132	107–133	107–133	
Median (IQR)	120	119	119	
Sex, *n* (%)				<2.2 × 10^–16^
Female	654 (36.4%)	3,224 (53.7%)	3,878 (49.7%)	
Male	1,144 (63.6%)	2,783 (46.3%)	3,927 (50.3%)	
Race, *n* (%)				<6.53 × 10^–4^
White	997 (55.5%)	3,324 (55.3%)	4,321 (55.4%)	
Black	293 (16.35%)	818 (13.6%)	111 (14.2%)	
Asian	21 (1.2%)	136 (2.3%)	157 (2.0%)	
Hispanic	325 (18.1%)	1,244 (20.7%)	1,569 (20.1%)	
Undetermined	6 (0.3%)	17 (0.3%)	23 (0.3%)	
Mixed/Others	156 (8.7%)	468 (7.8%)	624 (8.0%)	
Handedness, *n* (%)				3.46 × 10^–5^
Right	1,388 (77.2%)	4,912 (81.8%)	6,300 (80.7%)	
Left	137 (7.6%)	406 (6.8%)	543 (7.0%)	
Both	273 (15.2%)	689 (11.5%)	962 (12.3%)	
Highest parental education, *n* (%)				1.91 × 10^–3^
No highschool degree	96 (5.3%)	345 (5.7%)	441 (5.7%)	
Highschool/General educational development	139 (7.7%)	482 (8.0%)	621 (8.0%)	
Some college/Associate degree	534 (29.7%)	1,439 (24.0%)	1,973 (25.3%)	
Bachelor’s degree	483 (26.9%)	1,559 (26.0%)	2,042 (26.2%)	
Postgraduate	543 (30.2%)	2,177 (36.2%)	2,720 (34.8%)	
Undetermined	3 (0.2%)	5 (0.1%)	8 (0.1%)	

### 3.2. ADHD and cortical morphology

**FIGURE 2:**
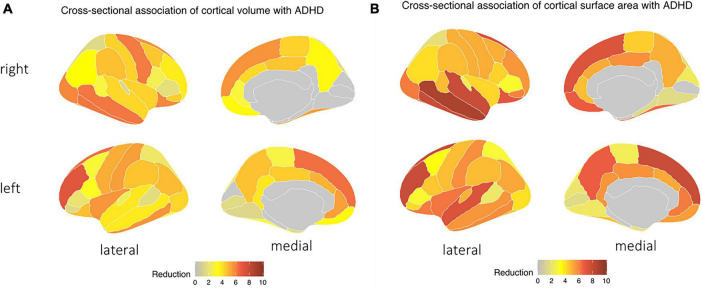
Children with ADHD tend to have a decrease in both in **(A)** cortical surface area and **(B)** cortical volume, particularly in the temporal and frontal lobes, as indicated by the blue area – independent of age, biological sex, race, handedness, and highest parental education as a marker of socioeconomic status, and after correction for multiple comparisons.

Children with ADHD had reduced cortical surface area and volume compared to those without – especially in frontal and temporal lobes (Figure 2). The presence of ADHD was associated with lower cortical surface area with most pronounced differences in the right middle-temporal gyrus (adjusted *p*-value = 1.574 × 10^–9^), left rostral-middle-frontal gyrus (adjusted *p*-value = 1.475 × 10^–8^), left superior frontal gyrus (adjusted *p*-value = 1.475 × 10^–8^), and right inferior/superior temporal gyrus (both adjusted *p*-value = 1.475 × 10^–8^). An ADHD diagnosis was also associated with reduced cortical volumes (Figure 2B), most pronounced in the left rostral middle-frontal gyrus (adjusted *p*-value = 8.044 × 10^–8^), left superior frontal gyrus (adjusted *p*-value = 5.107 × 10^–7^), right middle-temporal gyrus (adjusted *p*-value = 6.347 × 10^–7^), and the right inferior-temporal gyrus (adjusted *p*-value = 6.347 × 10^–7^) regions. No differences in sulcal depths and cortical thickness remained significant after FDR correction [see section “3.1. Structural MRI (Supplementary material)”].

### 3.3. ADHD and WM microstructure

**FIGURE 3:**
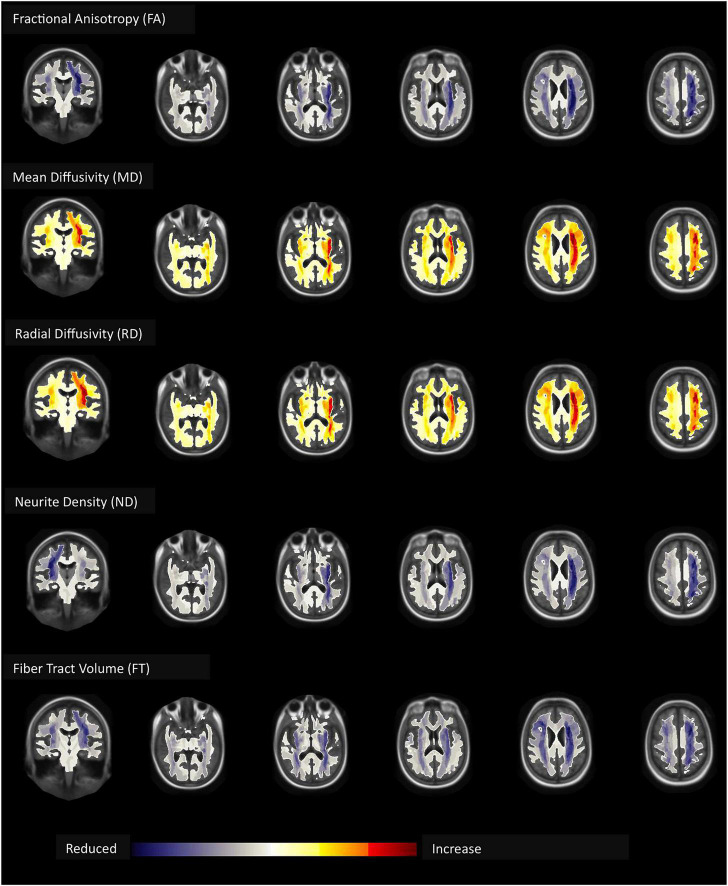
Children with ADHD compared to those without had lower fractional anisotropy (FA), neurite density (ND), and fiber tract (FT) volume but higher mean (MD) and radial (RD) diffusivity – most pronounced in the frontal and parietal white matter – independent of age, biological sex, race, handedness, and highest parental education as a marker of socioeconomic status, and after correction for multiple comparisons.

Children with ADHD had a significantly lower FA and ND, but higher MD and RD compared to children without ADHD – especially in frontal and parietal WM [see section “3.2. Diffusion MRI (Supplementary material)”]. In addition, ADHD was associated with lower FT. The highly significant changes (*p* < 0.001) in DTI metrics and FT were most notable in the corticostriatal tract which connects the superior frontal and parietal cortex with the striatum; WM tract connecting inferior frontal cortex and superior frontal cortex; and superior longitudinal fasciculus connecting parietal and frontal lobe (Figure 3). There were no differences in WM AD between children with and without ADHD diagnosis.

### 3.4. ADHD and functional connectivity

**FIGURE 4:**
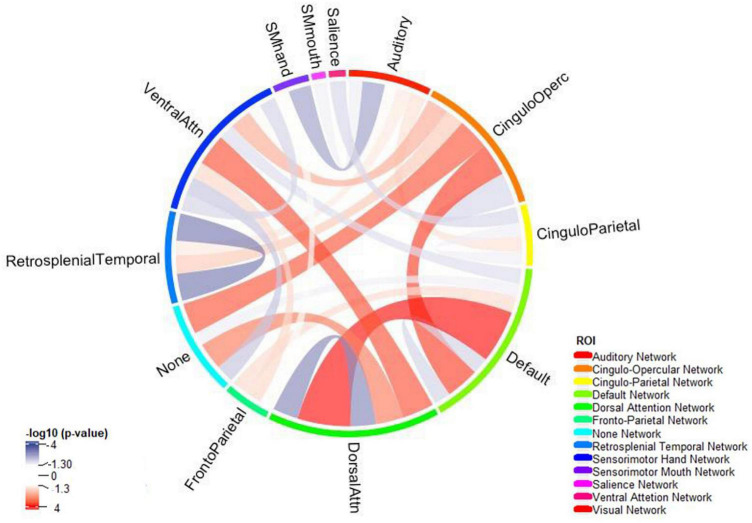
The connectogram summarizes reduced (blue) and increased (red) network connectivity between functional areas among children with ADHD compared to those without – independent of age, biological sex, race, handedness, and highest parental education as a marker of socioeconomic status, and after correction for multiple comparisons. Functional network was split using Gordon Parcellation.

Children with ADHD showed increased connectivity between the default network and the dorsal attention network (adjusted *p*-value = 3.721 × 10^–6^) and the cingulo-opercular network (adjusted *p*-value = 3.981 × 10^–4^) (Figure 4). Moreover, the correlation between the dorsal and the ventral attention networks (adjusted *p*-value = 5.324 × 10^–4^) was also higher among those with ADHD. In contrast, ADHD was associated with decreased intra-network connectivity of the dorsal attention (adjusted *p*-value = 2.169 × 10^–4^), default mode (adjusted *p*-value = 0.020), and retrosplenial temporal (adjusted *p*-value = 2.169 × 10^–4^) networks [see section “3.3. Rest-state fMRI (Supplementary material)”].

### 3.5. Machine learning classifiers for predicting ADHD

**FIGURE 5:**
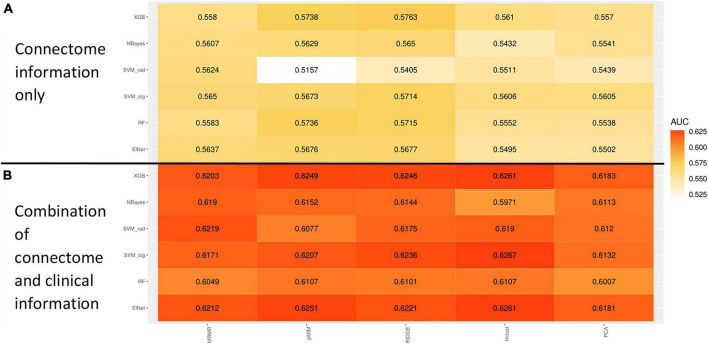
Heatmap showing the mean receiver operating characteristics (ROC) area under curve (AUC) across 100 validation folds from combination of different machine-learning classifiers and feature selection methods using imaging variables alone **(A)** or combination of clinical and imaging variables **(B)**.

The rate of ADHD diagnosis in both training/cross-validation (1,420 out of 6,244, 22.8%) and independent validation test (378 out of 1,561, 24.2%) sets were similar (*p* = 0.229). Table 2 summarize the demographics of subjects in training and independent validation sets. The Figure 5A heatmap depicts the mean AUCs across validation folds from five repeats of 10-fold cross-validations with neuroimaging variables alone (without any clinical information) as input. The combination of eXtreme Gradient Boosting (XGB) and RIDGE regularized logistic regression (RIDGE) for feature selection had the highest averaged AUC of 0.58. For combined input of clinical and neuroimaging variables, the best performance was achieved by the combination of Support Vector machine with radial kernel (SVM_rad) in combination with Hierarchical Clustering (HClust) feature selection with mean AUC of 0.627 (Figure 5B). In this model all clinical features (i.e., sex, age, race, highest parental education, and handedness) were selected by HClust. Selected feature with the feature importance can be found in section “2.4. Selected feature in final model (Supplementary material).” In the independent validation test set, the best performing model using neuroimaging variables achieved an AUC of 0.576 (95% CI = 0.546–0.610) with sensitivity of 56.61% and specificity of 64.86% compared to a model using clinical and neuroimaging variables which achieved AUC of 0.613 (95% CI = 0.580–0.645), with sensitivity of 60.05% and specificity of 56.47%. However, the difference between models using neuroimaging versus combined variables for prediction of ADHD diagnosis in the independent validation test cohort was not statistically significant (*p* = 0.0851).

**TABLE 2:** Demographic characteristics of train/Cross-validation versus independent test cohorts.

Patients’ characteristic in training/Cross-validation cohort and independent validation cohort			
	Training/Cross-validation cohort	Independent validation cohort	*p-*value training vs. independent
	6,244	1,561	
Patients with ADHD diagnosis	1,420 (22.7)	378 (24.2)	0.229
**Age**			0.930
Mean (SD)	119.2 (7.5)	119.6 (7.6)	
Min–max	107–133	107–132	
Median (IQR)	119	120	
**Sex, *n* (%)**			
Female	3,118(49.9)	760 (48.7)	0.292
Male	3,126 (50.0)	801 (51.3)	0.393
**Race, *n* (%)**			
White	3,470 (55.6)	851 (54.3)	0.470
Black	901 (14.4)	210 (13.5)	0.343
Asian	130 (2.0)	27 (1.3)	0.422
Hispanic	1,241 (19.9)	328 (21.0)	0.333
Undetermined	17 (0.3)	6 (0.4)	0.639
Mixed/Others	485 (7.8)	139 (8.9)	0.153
**Handedness, *n* (%)**			
Right	5,048 (80.8)	1,252 (80.2)	0.591
Left	440 (7.1)	103 (6.6)	0.477
Both	756 (12.1)	206 (13.2)	0.260
**Highest parental education, *n* (%)**			
No highschool degree	338 (5.4)	103 (6.6)	0.080
Highschool/General educational development	503 (8.1)	118 (7.6)	0.551
Some college/Associate degree	1,596 (25.6)	377 (24.2)	0.266
Bachelor’s degree	1,619 (25.9)	423 (27.1)	0.364
Postgraduate	2,182 (34.9)	538 (34.5)	0.744
Undetermined	6 (0.1)	2 (0.1)	1.000

## 4. Discussion

Leveraging a large population dataset, we identified the multimodal neuroimaging correlates of ADHD in 9–10-year-old children. While many prior studies have examined smaller selective cohorts of ADHD children versus typically developing controls, we adopted more inclusive criteria comparing multimodal neuroimaging correlates of those with and without ADHD diagnosis in a large demographically representative population of American children. By having a large sample size, our study approach highlights the neuroimaging correlates of ADHD in demographically representative cohort. Our study population are more representative of all-comers children presenting to clinical practices, when ADHD is often accompanied by other comorbidities. We found that children with ADHD had WM microstructural disintegrity and reduced neural density (particularly in frontal and parietal lobes), decreased cortical volume and surface area (most striking in the frontal and temporal lobes); and altered connectivity of attention functional networks compared to those without – after correction for children’s age, sex, race/ethnicity, handedness, and highest parental education (as a marker of socioeconomic status). Our findings highlight the neurobiological and connectome mechanism of ADHD among preadolescent children at demographically representative population-level. In addition, we showed the potentials and limitations of machine learning classifiers in combining neuroimaging metrics (and clinical variables) to predict ADHD diagnosis. In addition, multimodal framework of our study further highlights the role of frontal lobe cortical regions and associated WM tracts in neurobiology of childhood ADHD (Bernanke et al., 2022).

Children with ADHD have been found to have morphological differences in brain structure compared to typically developing children. Our study shows that childhood ADHD is associated with reduced cortical volumes and surface areas in the frontal lobe, cingulate, and temporal lobe. These areas are known to be associated with complex cognitive behaviors such as decision-making, reasoning, personality expression, and maintaining social appropriateness (El-Baba and Schury, 2022) and visuo-attentional processes (Zarka et al., 2021). Prior studies have proposed an association between smaller prefrontal cortex volume in ADHD children and their ability to suppress their responses to salient but otherwise irrelevant events (Casey et al., 1997). Diffusion MRI results complement our morphological findings as children with ADHD also had lower FA, FT, and ND but higher MD and RD in WM tracts originating from the frontal cortex compatible with reduced microstructural integrity and neuronal density, which can affect attention, school function, and non-verbal intelligence (De Zeeuw et al., 2012; Tung et al., 2021).

Furthermore, functional connectivity is also perturbed in individuals with ADHD (Cortese et al., 2018; Gao et al., 2019; Sutcubasi et al., 2020). In our study, children with ADHD had reduced intra-connectivity in the dorsal attention network, the default-mode network and the retrosplenial network. The dorsal attention network as part of task positive network is active while focusing on a particular task and it is important for processing of relevant information and filter out irrelevant information (Rohr et al., 2017). Of note, parts of the dorsal attention network are within the middle temporal region which we prior identify as a region with reduced cortical volume and sulcal depths. On the other hand, default mode network is active while the patient is engaging in internally focused tasks (Buckner, 2013). According to the default-mode network hypothesis, lapses in attention, among ADHD children, are caused by spontaneous intrusions of this network (Sonuga-Barke and Castellanos, 2007). Recent neuroanatomical models localize parts of the default-mode network to prefrontal region and cingulum (Alves et al., 2019). We found that although the intra-connectivity of both dorsal attention and default-mode networks decreases, their network interconnectivity increase implying an overstimulation between these two networks (Figure 4). A recent study confirms these findings reporting hyperconnectivity of the default-mode with task-relevant networks (Duffy et al., 2021). Association of ADHD with default mode network dysconnectivity was also reported in a meta-analysis of 21 studies with 700 ADHD patients and 580 controls (Gao et al., 2019), and a meta-analysis of 20 studies with 944 ADHD patients and 1,121 controls (Sutcubasi et al., 2020). Of note, our morphological and microstructural findings in frontal and temporal lobes may – at least partially – explain aberrations of functional connectivity among ADHD children, especially in dorsal attention and default-mode networks.

In contrast to several other studies, we found no significant association between ADHD diagnosis and cortical thickness (unlike cortical volume). Possible explanation could be: both surface area and cortical thickness play a significant mediating role in determining diagnostic differences in volume, with regional brain variation, the contribution of surface area might be bigger than thickness and by using multiple testing correcting the effect of thickness was not statistically significant. Of note, Shaw et al. (2007) reported a maturation delay in reaching peak cortical thickness in children with ADHD. Different cortical and subcortical regions have been implicated in pathogenesis of ADHD: the left parahippocampal gyrus (Carmona et al., 2005), occipital associated cortices (Narr et al., 2009), amygdala and nuclei accumbens (Hoogman et al., 2017; Boedhoe et al., 2020). The difference between our study and priors may be due to selection bias, age-related differences, or sociodemographic variations (Hoogman et al., 2019).

Neuroimaging studies may also facilitate ADHD diagnosis in children. Currently, clinical interviews with children, parents, and caregivers are mainstays of ADHD diagnosis in children (Wolraich et al., 2019). However, the collected questionnaires are subjective and sometimes inconsistent (Gualtieri and Johnson, 2005). Thus, quantitative objective methodologies can potentially supplement clinical examination to achieve a more reliable diagnosis particularly in presence of cultural, language, or communication barriers. This is particularly important since delayed diagnosis of ADHD is associated with higher risk of developing mood and anxiety disorders, substance abuse, and/or personality disorders (Katzman et al., 2017). In our study, we evaluated the applicability of machine learning classifiers in prediction of ADHD diagnosis using multimodal neuroimaging metrics. By exploiting the multimodal data more information for individual subject can be gathered and provide the chance of finding missing links in complex mental illness and giving a comprehensive overview of the same cohort.

Using a rigorous cross-validation framework we could minimize the risk of overfitting and ensure the stability of the model. The stability of our best performed model was proofed with our independent validation set, which was isolated from training and optimization process. A machine-learning model using neuroimaging metrics alone could achieve an AUC of 0.576 (95% CI = 0.546–0.610), which was increased to 0.613 (95% CI = 0.580–0.645) by adding clinical variables, although not statistically different (*p* = 0.0851). To mitigate the issue of class imbalance in our study (with a 1:3 ratio of children with and without ADHD), we employed the AUC of ROC as the primary measure of performance, as it is not affected by class imbalance, unlike classification accuracy. Prior studies have reported higher accuracy of machine-learning models for prediction of ADHD; however, they have used highly selective ADHD versus control subjects (Ghiassian et al., 2016; Sen et al., 2018), or lacked true independent validation cohort (Deshpande et al., 2015; Qureshi et al., 2017). While our results show that prediction is possible in a diverse group, it still needs to be optimized, e.g., inclusion of clinical features. We demonstrate both the potentials and limitations of machine-learning classifiers for prediction of ADHD diagnosis in general population using multimodal neuroimaging metrics.

The main strength of our study is large cohort of children from a narrow age-group, who are reflective of demographically diverse U.S. population. Indeed, one of the objectives of ABCD study is to elucidate factors that influence the course of mental illness (Jernigan and Brown, 2018), which also reflects our aim to investigate microstructural, morphological, and functional connectivity correlates of ADHD in preadolescent children. At the same time, our analysis was limited by using processed imaging metrics from ABCD Consortium. Although a standardized protocol was used for harmonized image acquisition, site differences might still influence our analysis. In addition, different brain atlases were used for sMRI (Desikan-Killiany atlas), dMRI (Atlas Track atlas), and fMRI (Gordon Network Parcellation). It is important to note that these atlases may have variations in the number and location of ROI partitions, which complicates the understanding of the connecting between structure and function. Our present study focused on cortical volume with a few subcortical regions due to the natural noisiness of subcortical measurements. In addition, WM tracts thickness and microstructure provided complementary information for evaluation of whole brain. Nevertheless, some studies suggested that subcortical regions also show ADHD-dependent variations (Hoogman et al., 2017; Boedhoe et al., 2020). The inclusion of this information contains the potential to better understand the pathological mechanisms. Additionally, the linear regression method we employed may not be able to detect more intricate relationships. Further limitations of our study were the absence of cognitive parameters associated with ADHD and separation of patients into subtypes. The diagnosis was generated by K-SADS questionnaires at different sites with various levels of expertise and there is no diagnostic validation study nested with the ABCD study. Finally, we were not able to analyze the effects ADHD subtypes, duration of diagnosis, or any treatment.

## 5. Conclusion

We could identify the neurostructural and -functional correlates of ADHD in demographically representative cohort of American children. The comprehensive and convergent results implicated brain regions and networks involved in impulse control, executive function, and concentration in pathogenesis of childhood ADHD. Specifically, our results showed the association of childhood ADHD with frontal lobe cortical volume reduction and lower WM integrity and neuronal density. These morphological and microstructural findings may also explain the observed aberrations of functional connectivity in dorsal attention and default-mode networks among children with ADHD. We also showed limited potentials of machine-learning classifiers in prediction of ADHD diagnosis at general population level. Nevertheless, these neuroimaging correlates can potentially help with diagnosis, treatment monitoring, and response prediction.

## Data availability statement

Publicly available datasets were analyzed in this study. This data can be found here: the datasets Adolescent Brain Cognitive Development Study: https://nda.nih.gov/abcd/.

## Ethics statement

The studies involving human participants were reviewed and approved by the Ethics Committee of ABCD. Written informed consent to participate in this study was provided by the participants’ legal guardian/next of kin.

## Author contributions

HL implemented the analysis, interpreted the findings, and wrote and revised the manuscript. SP designed the study, interpreted the findings, and supported the writing and revision of the manuscript. SH assisted in data analysis. AMo, SK, AMa, RC, DS, LM, and KK assisted in interpretation the findings and reviewed the manuscript. All authors contributed to the article and approved the submitted version.

## Footnotes

1
https://abcdstudy.org/wp-content/uploads/2021/05/ABCD_Website_MRI_Acq.pdf

2
https://abcdstudy.org/images/Protocol_Imaging_Sequences.pdf

## References

[B1] Alves P. N. Foulon C. Karolis V. Bzdok D. Margulies D. S. Volle E. (2019). An improved neuroanatomical model of the default-mode network reconciles previous neuroimaging and neuropathological findings. *Commun. Biol.* 2:370. 10.1038/s42003-019-0611-3 31633061 PMC6787009

[B2] Aoki Y. Cortese S. Castellanos F. X. (2018). Research review: Diffusion tensor imaging studies of attention-deficit/hyperactivity disorder: Meta-analyses and reflections on head motion. *J. Child Psychol. Psychiatry* 59 193–202. 10.1111/jcpp.12778 28671333

[B3] Bernanke J. Luna A. Chang L. Bruno E. Dworkin J. Posner J. (2022). Structural brain measures among children with and without ADHD in the adolescent brain and cognitive development study cohort: A cross-sectional US population-based study. *Lancet Psychiatry* 9 222–231. 10.1016/S2215-0366(21)00505-8 35143759

[B4] Boedhoe P. S. W. Van Rooij D. Hoogman M. Twisk J. W. R. Schmaal L. Abe Y. (2020). Subcortical brain volume, regional cortical thickness, and cortical surface area across disorders: Findings from the ENIGMA ADHD, ASD, and OCD working groups. *Am. J. Psychiatry* 177 834–843. 10.1176/appi.ajp.2020.19030331 32539527 PMC8296070

[B5] Buckner R. L. (2013). The brain’s default network: Origins and implications for the study of psychosis. *Dialogues Clin. Neurosci.* 15 351–358. 10.31887/DCNS.2013.15.3/rbuckner 24174906 PMC3811106

[B6] Carmona S. Vilarroya O. Bielsa A. Tremols V. Soliva J. C. Rovira M. (2005). Global and regional gray matter reductions in ADHD: A voxel-based morphometric study. *Neurosci. Lett.* 389 88–93. 10.1016/j.neulet.2005.07.020 16129560

[B7] Casey B. J. Cannonier T. Conley M. I. Cohen A. O. Barch D. M. Heitzeg M. M. (2018). The adolescent brain cognitive development (ABCD) study: Imaging acquisition across 21 sites. *Dev. Cogn. Neurosci.* 32 43–54. 10.1016/j.dcn.2018.03.001 29567376 PMC5999559

[B8] Casey B. J. Castellanos F. X. Giedd J. N. Marsh W. L. Hamburger S. D. Schubert A. B. (1997). Implication of right frontostriatal circuitry in response inhibition and attention-deficit/hyperactivity disorder. *J. Am. Acad. Child Adolesc. Psychiatry* 36 374–383. 10.1097/00004583-199703000-00016 9055518

[B9] Chiang H.-L. Tseng W.-Y. I. Wey H.-Y. Gau S. S.-F. (2020). Shared intrinsic functional connectivity alterations as a familial risk marker for ADHD: A resting-state functional magnetic resonance imaging study with sibling design. *Psychol. Med.* 52 1736–1745. 10.1017/s0033291720003529 33046145

[B10] Cortese S. Adamo N. Del Giovane C. Mohr-Jensen C. Hayes A. J. Carucci S. (2018). Comparative efficacy and tolerability of medications for attention-deficit hyperactivity disorder in children, adolescents, and adults: A systematic review and network meta-analysis. *Lancet Psychiatry* 5 727–738. 10.1016/S2215-0366(18)30269-4 30097390 PMC6109107

[B11] Danielson M. L. Bitsko R. H. Ghandour R. M. Holbrook J. R. Kogan M. D. Blumberg S. J. (2018). Prevalence of parent-reported ADHD diagnosis and associated treatment among U.S. children and adolescents, 2016. *J. Clin. Child Adolesc. Psychol.* 47 199–212. 10.1080/15374416.2017.1417860 29363986 PMC5834391

[B12] De Zeeuw P. Weusten J. Van Dijk S. Van Belle J. Durston S. (2012). Deficits in cognitive control, timing and reward sensitivity appear to be dissociable in ADHD. *PLoS One* 7:e51416. 10.1371/journal.pone.0051416 23236497 PMC3517570

[B13] Deshpande G. Wang P. Rangaprakash D. Wilamowski B. (2015). Fully connected cascade artificial neural network architecture for attention deficit hyperactivity disorder classification from functional magnetic resonance imaging data. *IEEE Trans. Cybern.* 45 2668–2679. 10.1109/TCYB.2014.2379621 25576588

[B14] Desikan R. S. Ségonne F. Fischl B. Quinn B. T. Dickerson B. C. Blacker D. (2006). An automated labeling system for subdividing the human cerebral cortex on MRI scans into gyral based regions of interest. *NeuroImage* 31 968–980. 10.1016/j.neuroimage.2006.01.021 16530430

[B15] Duffy K. A. Rosch K. S. Nebel M. B. Seymour K. E. Lindquist M. A. Pekar J. J. (2021). Increased integration between default mode and task-relevant networks in children with ADHD is associated with impaired response control. *Dev. Cogn. Neurosci.* 50:100980. 10.1016/j.dcn.2021.100980 34252881 PMC8278154

[B16] El-Baba R. M. Schury M. P. (2022). *Neuroanatomy, frontal cortex.* Treasure Island, FL: StatPearls. 32119370

[B17] Gao Y. Shuai D. Bu X. Hu X. Tang S. Zhang L. (2019). Impairments of large-scale functional networks in attention-deficit/hyperactivity disorder: A meta-analysis of resting-state functional connectivity. *Psychol. Med.* 49 2475–2485. 10.1017/S003329171900237X 31500674

[B18] Garavan H. Bartsch H. Conway K. Decastro A. Goldstein R. Z. Heeringa S. (2018). Recruiting the ABCD sample: Design considerations and procedures. *Dev. Cogn. Neurosci.* 32 16–22. 10.1016/j.dcn.2018.04.004 29703560 PMC6314286

[B19] Ghiassian S. Greiner R. Jin P. Brown M. R. (2016). Using functional or structural magnetic resonance images and personal characteristic data to identify ADHD and Autism. *PLoS One* 11:e0166934. 10.1371/journal.pone.0166934 28030565 PMC5193362

[B20] Gordon E. M. Laumann T. O. Adeyemo B. Huckins J. F. Kelley W. M. Petersen S. E. (2016). Generation and evaluation of a cortical area parcellation from resting-state correlations. *Cereb. Cortex* 26 288–303. 10.1093/cercor/bhu239 25316338 PMC4677978

[B21] Gualtieri C. T. Johnson L. G. (2005). ADHD: Is objective diagnosis possible? *Psychiatry* 2 44–53. PMC2993524 21120096

[B22] Hagler D. J. Ahmadi M. E. Kuperman J. Holland D. Mcdonald C. R. Halgren E. (2009). Automated white-matter tractography using a probabilistic diffusion tensor atlas: Application to temporal lobe epilepsy. *Hum. Brain Mapp.* 30 1535–1547. 10.1002/hbm.20619 18671230 PMC2754725

[B23] Hagler D. J. Hatton S. Cornejo M. D. Makowski C. Fair D. A. Dick A. S. (2019). Image processing and analysis methods for the adolescent brain cognitive development study. *NeuroImage* 202:116091. 10.1016/j.neuroimage.2019.116091 31415884 PMC6981278

[B24] Haider S. P. Mahajan A. Zeevi T. Baumeister P. Reichel C. Sharaf K. (2020). PET/CT radiomics signature of human papilloma virus association in oropharyngeal squamous cell carcinoma. *Eur. J. Nucl. Med. Mol. Imaging* 47 2978–2991. 10.1007/s00259-020-04839-2 32399621

[B25] Hoogman M. Bralten J. Hibar D. P. Mennes M. Zwiers M. P. Schweren L. S. J. (2017). Subcortical brain volume differences in participants with attention deficit hyperactivity disorder in children and adults: A cross-sectional mega-analysis. *Lancet Psychiatry* 4 310–319. 10.1016/S2215-0366(17)30049-4 28219628 PMC5933934

[B26] Hoogman M. Muetzel R. Guimaraes J. P. Shumskaya E. Mennes M. Zwiers M. P. (2019). Brain imaging of the cortex in ADHD: A coordinated analysis of large-scale clinical and population-based samples. *Am. J. Psychiatry* 176 531–542. 10.1176/appi.ajp.2019.18091033 31014101 PMC6879185

[B27] Jernigan T. L. Brown S. A. (2018). Introduction. *Dev. Cogn. Neurosci.* 32 1–3. 10.1016/j.dcn.2018.02.002 29496476 PMC6969247

[B28] Katzman M. A. Bilkey T. S. Chokka P. R. Fallu A. Klassen L. J. (2017). Adult ADHD and comorbid disorders: Clinical implications of a dimensional approach. *BMC Psychiatry* 17:302. 10.1186/s12888-017-1463-3 28830387 PMC5567978

[B29] Kaufman J. Birmaher B. Brent D. Rao U. Flynn C. Moreci P. (1997). Schedule for affective disorders and schizophrenia for school-age children-present and lifetime version (K-SADS-PL): Initial reliability and validity data. *J. Am. Acad. Child Adolesc. Psychiatry* 36 980–988. 10.1097/00004583-199707000-00021 9204677

[B30] Kumar U. Arya A. Agarwal V. (2017). Neural alterations in ADHD children as indicated by voxel-based cortical thickness and morphometry analysis. *Brain Dev.* 39 403–410. 10.1016/j.braindev.2016.12.002 28057397

[B31] Narr K. L. Woods R. P. Lin J. Kim J. Phillips O. R. Del’homme M. (2009). Widespread cortical thinning is a robust anatomical marker for attention-deficit/hyperactivity disorder. *J. Am. Acad. Child Adolesc. Psychiatry* 48 1014–1022. 10.1097/CHI.0b013e3181b395c0 19730275 PMC2891193

[B32] Qureshi M. N. I. Oh J. Min B. Jo H. J. Lee B. (2017). Corrigendum: Multi-modal, multi-measure, and multi-class discrimination of ADHD with hierarchical feature extraction and extreme learning machine using structural and functional brain MRI. *Front. Hum. Neurosci.* 11:292. 10.3389/fnhum.2017.00292 28579953 PMC5450098

[B33] Rohr C. S. Vinette S. A. Parsons K.a.L Cho I. Y. K. Dimond D. Benischek A. (2017). Functional connectivity of the dorsal attention network predicts selective attention in 4-7 year-old girls. *Cereb. Cortex* 27 4350–4360. 27522072 10.1093/cercor/bhw236

[B34] Sen B. Borle N. C. Greiner R. Brown M. R. G. (2018). A general prediction model for the detection of ADHD and Autism using structural and functional MRI. *PLoS One* 13:e0194856. 10.1371/journal.pone.0194856 29664902 PMC5903601

[B35] Shaw P. Eckstrand K. Sharp W. Blumenthal J. Lerch J. P. Greenstein D. (2007). Attention-deficit/hyperactivity disorder is characterized by a delay in cortical maturation. *Proc. Natl. Acad. Sci. U.S.A.* 104 19649–19654. 18024590 10.1073/pnas.0707741104 PMC2148343

[B36] Sonuga-Barke E. J. Castellanos F. X. (2007). Spontaneous attentional fluctuations in impaired states and pathological conditions: A neurobiological hypothesis. *Neurosci. Biobehav. Rev.* 31 977–986. 10.1016/j.neubiorev.2007.02.005 17445893

[B37] Sudre G. Gildea D. E. Shastri G. G. Sharp W. Jung B. Xu Q. (2022). Mapping the cortico-striatal transcriptome in attention deficit hyperactivity disorder. *Mol. Psychiatry*. 28, 792–800. 10.1038/s41380-022-01844-9 36380233 PMC9918667

[B38] Sutcubasi B. Metin B. Kurban M. K. Metin Z. E. Beser B. Sonuga-Barke E. (2020). Resting-state network dysconnectivity in ADHD: A system-neuroscience-based meta-analysis. *World J. Biol. Psychiatry* 21 662–672. 10.1080/15622975.2020.1775889 32468880

[B39] Tung Y. H. Lin H. Y. Chen C. L. Shang C. Y. Yang L. Y. Hsu Y. C. (2021). Whole brain white matter tract deviation and idiosyncrasy from normative development in Autism and ADHD and unaffected siblings link with dimensions of psychopathology and cognition. *Am. J. Psychiatry* 178 730–743. 10.1176/appi.ajp.2020.20070999 33726525

[B40] Wolraich M. L. Hagan J. F. Allan C. Chan E. Davison D. Earls M. (2019). Clinical practice guideline for the diagnosis, evaluation, and treatment of attention-deficit/hyperactivity disorder in children and adolescents. *Pediatrics* 144:e20192528. 10.1542/peds.2019-2528 31570648 PMC7067282

[B41] Wu W. Mcanulty G. Hamoda H. M. Sarill K. Karmacharya S. Gagoski B. (2020). Detecting microstructural white matter abnormalities of frontal pathways in children with ADHD using advanced diffusion models. *Brain Imaging Behav.* 14 981–997. 10.1007/s11682-019-00108-5 31041662

[B42] Yang R. Jernigan T. (2020). *Adolescent brain cognitive development study (ABCD) – annual release 3.0.* Available online at: https://nda.nih.gov/study.html?id=1042

[B43] Zarka D. Leroy A. Cebolla A. M. Cevallos C. Cheron G. (2021). Neural generators involved in visual cue processing in children with attention-deficit/hyperactivity disorder (ADHD). *Eur. J. Neurosci.* 53 1207–1224. 10.1111/ejn.15040 33169431

