# Supplementary material for: Population level multimodal neuroimaging correlates of attention-deficit hyperactivity disorder among children
Source: Front Neurosci. 2023 Feb 22;17:1138670. doi: 10.3389/fnins.2023.1138670 (PMC9992191; doi:10.3389/fnins.2023.1138670)
Supplement: Supplementary file 1 [file Data_Sheet_1.docx]

**Supplemental Material**

1. **List of Atlas 1**
   1. AtlasTrack Atlas 1
   2. Desikan-Killiany Atlas 1
   3. Gordon Network Parcellation 2
2. **Machine Learning 3**
   1. Classifier 3
   2. Elastic Net Regularization 3
   3. Random Forest 3
   4. Support Vector Machine 3
   5. Naïve Bayes3
   6. Extreme Gradient Boosting 3
   7. Feature Selection Algorithm 4
   8. Hierarchical Clustering 4
   9. Minimal Redundancy Maximal Relevance 4
   10. Pearson correlation-based redundancy reduction with Mutual Information 4
   11. Principal Component Analysis – based feature selection 4
   12. Logistical Regression using Ridge regularization 5
   13. Hyperparameter bounds for Bayesian optimization 6
   14. Selected feature in final model 6
3. **Multimodal Multiple Logistic Regression 9**
   1. Structural MRI 9
   2. Diffusion MRI 12
   3. Rest-state fMRI 16
4. **References 18**

**1. List of Atlas Regions**

**1.1. AtlasTrack**

R/L fornix

R/L cingulum (cingulate)

R/L cingulum (parahippocampal)

R/L corticospinal tract or pyramidal tract

R/L anterior thalamic radiations

R/L uncinate fasciculus

R/L inferior longitudinal fasciculus

R/L inferior fronto-occipital fasciculus

foreceps major

foreceps minor

corpus callosum

R/L superior longitudinal fasciculus

R/L temporal superior longitudinal fasciculus

R/L parietal superior longitudinal fasciculus

R/L optic radiation

R/L superior corticostriate

R/L superior corticostriate (frontal)

R/L superior corticostriate (parietal)

R/L striatal inferior frontal cotex

R/L inferior frontal superior frontal cortex

R/L fornix; excluding fimbria

All Fibers

R/L hemisphere Fibers no CC

R/L hemisphere Fibers

**1.2. Desikan-Killiany atlas**

R/L Banks of Superior Temporal Sulcus

R/L caudal anterior cingulate

R/L caudal middle frontal

R/L cuneus

R/L entorhinal

R/L fusiform

R/L inferior parietal

R/L inferior temporal

R/L isthmus cingulate

R/L lateral occipital

R/L lateral orbitofrontal

R/L lingual

R/L medial orbitofrontal

R/L middle temporal

R/L parahippocampal

R/L paracentral

R/L pars opercularis

R/L pars orbitalis

R/L pars triangularis

R/L pericalcarine

R/L postcentral

R/L posterior cingulate

R/L precentral

R/L precuneus

R/L rostral anterior cingulate

R/L rostral middle frontal

R/L superior frontal

R/L superior parietal

R/L superior temporal

R/L supramarginal

R/L frontal pole

R/L temporal pole

R/L transverse temporal

R/L insula

**1.3. Gordon Network Parcellation**

Default Mode

Somatomotor-Hand

Somatomotor-Mouth

Visual

Fronto-Parietal

Auditory

Cingulo-Parietal

Retrosplenial-Temporal

Ventral Attention

Salience

Dorsal Attention

Singulo-Opercular

**2. Machine Learning**

The dimensionality reduction and machine learning framework applied here are similar to Haider et al.’s work (Haider et al., 2020) with modification from Weber et al.’s work (Weber et al., 2022). In the following, descriptions of the algorithms used are replicated from the prior works:

**2.1. Classifier**

**2.1.1. Elastic Net Regularization (ElNet)**

Elastic Net linear regression performs variable selection in using Least Absolute Shrinkage and Selection Operator (LASSO) and Ridge techniques to regularize regression model. The mixing parameter alpha determine the weight in the combination of RIDGE and Lasso, which was tuned in Bayesian Optimization. Lamda was determined to maximize the mean cross-validated area under the curve in the internal 10-fold cross-validation. Elastic Net was implemented using cv.glmnet from the glmnet package in R (Friedman et al., 2010) .

**2.1.2. Random Forest (RF)**

The random forest model described by Breiman (Breiman, 2001) was built in R using the “randomForest” package (4.7-1.1). Using the command “randomForest” 1000 tres was configured to grow and perform sampling of cases with replacement. The “mtry” (number of features randomly sampled as candidates at each split) and “maxnodes” (maximum number of terminal nodes in a tree) parameters were tuned in Bayesian optimization. All other function parameters were kept in default.

**2.1.3. Support Vector Machine (SVM) with sigmoid (SVM sig) and radial (SVM rad) kernel**

SVM were implemented using R *e1071 (David Meyer, 2022)* package. In binary classification, SVM aim to identify the optimal separating hyperplane between two groups by maximizing the margins between the classes’ closest points. Cost refers to a parameter determining how the number of datapoints in the margin is penalized, hence, how wide the margin is.

*Gamma* and *cost* were treated as hyperparameters and tuned in Bayesian Optimization. All other parameters were set in default.

**2.1.4. Naïve Bayes (NBayes)**

We used naive_bayes in R naivebayes package (Majka, 2019) to create a model. Gaussian distribution was applied for all features, and no Laplace smoothing was applied. NBayes has no tunable hyperparameters other than feature count, which was tuned in Bayesian Optimization if applicable in respective combinations with feature selection algorithms.

**2.1.5. Extreme Gradient Boosting (XGB)**

XGB was implemented using gbtree option in R xgboost (Tianqi Chen, 2022) package in tree-booster mode. XGB is an improved implementation of gradient boosting decision trees that uses more accurate approximations to find the best tree in iterative least-squares regression. Contrarily to common gradient boosting, XGB uses advanced regularization and second-order gradients [9]. Specifications of XGB functionality are the learning rate eta that reflects on step size shrinkage; the required minimum loss reduction for each further partition gamma; the maximum depth of a tree max_depth; the minimum weight of each child tree min_child_weight; the subsample ratio of training instances applied in every boosting iteration subsample as well as of columns colsample_by_tree; and the regularization parameter lambda. All of the aforementioned parameters were tuned in Bayesian Optimization. The rest of parameters were set by the default package recommendation.

**2.2. Feature Selection Algorithms**

**2.2.1. Hierarchical Clustering (HClust)**

First, a “euclidean” feature distance matrix of all radiomics features was computed using the R “stats” package (version 3.6.0) (R Development Core Team, 2019) “dist” function, followed by hierarchical clustering by means of the “stats” “hclust” function using Ward clustering with Ward’s clustering criterion implemented (i.e. “ward.D2” package option) (Murtagh and Legendre, 2014). The dendrogram was cut until 20 clusters remained. We extracted “meta-features” by averaging all features in any remaining cluster. Hierarchical clustering was performed with (cross-validation) training data, and only the averaging operations were applied to all cases. The 20 resulting meta features were used for training and classification.

**2.2.2. Minimal Redundancy Maximal Relevance (MRMR)**

mRMR function in R praznik package (De Jay et al., 2013) was used for MRMR feature selection. The n most predictive feature was selected through Bayesion optimization. MRMR combines maximum relevance feature selection, which considers the correlation of each feature with the final outcome variable, with minimum redundancy, which is a measure of joint distribution and information gain.

**2.2.3. Pearson correlation-based redundancy reduction with Mutual InformationMaximization filter (pMIM)**

First, Pearson correlation between every possible feature pair was determined by using the R stats package. Second findCorrelation as specified in the R caret package (Kuhn, 2022) was then applied to find and exclude features of highest correlation. Subsequently, a mutual information filter was introduced using MIM function of the R mRMR package. N was treated as a hyperparameter and tuned in Bayesian Optimization.

**2.2.4. Principal Component Analysis (PCA)-based feature selection**

Singular value decomposition based principal component analysis was performed on the (cross-validation) training data using the R “stats” package (version 3.6.0) (R Development Core Team, 2019) “prcomp” function.. Following PCA-based feature selection as suggested by Song et al. We selected the n features contributing most to feature extraction. N was treated as a hyperparameter and optimized in Bayesian Optimization.

**2.2.5. Logistic regression using Ridge regularization (RIDGE)**

The glmnet function in R cv.glmnet (Friedman et al., 2010) package was used. The inherent 10-fold cross validation mode determined the lambda penalty when fitting a Ridge logistic regression to all features. Subsequently, the n features of highest regression coefficient value were selected, and n was treated as a hyperparameter and tuned in Bayesian Optimization.

**2.3. Hyperparameter bounds for Bayesian optimization**

The following parameters were selected based on Weber et al.’s, Avery et al.’s (Avery et al., 2022) and Haider et al.'s framework.

| **Machine Learning Classifiers** | | | | |
| --- | --- | --- | --- | --- |
| *Abbreviation* | *Classifier* | *Hyperparameters* | *Upper and Lower Bound* | *Repetitions in Bay. Opt.* |
| ElNet | Elastic Net | alpha | 0 – 1 | 100 |
|  |  | n (Features) | 2 – 30 | 200 |
| NBayes | Naive Bayes^a^ | n (Features) | 2 – 30 | 100 |
|  |  | n (Features) | 2 – 30 | 150 |
| RF | Random Forest | maxnodes | 2 – 2^15^ | 150 |
|  |  | mtry | 2 – 40 | 150 |
|  |  | n (Features) | 2 – 30 | 50 |
| SVM rad | SVM radial | Cost | 0.1 – 10 | 150 |
|  |  | gamma | 0 – 0.5 | 150 |
|  |  | n (Features) | 2 – 30 | 200 |
| SVM sig | SVM sigmoid | Coef0 | 0 – 1 | 200 |
|  |  | cost | 0.1 – 10 | 200 |
|  |  | gamma | 0 – 0.5 | 200 |
|  |  | n (Features) | 2 – 30 | 150 |
| XGB | Extreme Gradient Boosting | Eta | 0 – 1 | 200 |
|  |  | Gamma | 0 – 5 | 200 |
|  |  | max_depth | 5 – 15 | 200 |
|  |  | Min_child_weight | 0 – 20 | 200 |
|  |  | Subsample | 0.8 – 1 | 200 |
|  |  | Colsample_by_tree | 0.8 – 1 | 200 |
|  |  | lambda | 0.5 – 1 | 200 |

**2.4. Selected feature in final model**

**2.4.1. With connectome information and selected clinical information**

The following listed features are selected in our final model with Support Vector Machine with radial kernel and Hierarchical Clustering as feature selection method. Similar features where group in similar clusters.

| Rank | Feature |
| --- | --- |
| 1 | sex |
| 2 | interview_age |
| 3 | race_var |
| 4 | handedness |
| 5 | education |
| 6 | Cluster_1 |
| 7 | Cluster_2 |
| 8 | Cluster_3 |
| 9 | Cluster_4 |
| 10 | Cluster_5 |
| 11 | Cluster_6 |
| 12 | Cluster_7 |
| 13 | Cluster_8 |
| 14 | Cluster_9 |
| 15 | Cluster_10 |
| 16 | Cluster_11 |
| 17 | Cluster_12 |
| 18 | Cluster_13 |
| 19 | Cluster_14 |
| 20 | Cluster_15 |
| 21 | Cluster_16 |
| 22 | Cluster_17 |
| 23 | Cluster_18 |
| 24 | Cluster_19 |
| 25 | Cluster_20 |

**2.4.2. With connectome information only**

Using the “xgb.importance” function from the xgboost package (version 0.6.4.1) (Tianqi Chen, 2021) a score quantifying the total gain of each feature was reported. The feature importance was rank using the “gain”.

| **Modality** | **Region** | **Feature name** | **Feature importance** | |
| --- | --- | --- | --- | --- |
|  |  |  | **Rank** | **Score** |
| **rsfMRI** | Average correlation between cingulo-opercular network and ventral attention network | rsfMRI_rsfmri_c_ngd_cgc_ngd_vta | **1** | 0.0605 |
| **DTI** | Fiber tract volume in DTI atlas tract left uncinate | DTI_dmdtifp1_180 | **2** | 0.0484 |
| **sMRI** | Cortical area in APARC ROI rh-middletemporal | sMRI_smri_area_cdk_mdtmrh | **3** | 0.0469 |
| **rsfMRI** | Average correlation between cingulo-opercular network and ventral attention network | rsfMRI_rsfmri_c_ngd_ad_ngd_smh | **4** | 0.0467 |
| **rsfMRI** | Average correlation between \"none\" network and ventral attention network | rsfMRI_rsfmri_c_ngd_n_ngd_vta | **5** | 0.0458 |
| **rsfMRI** | Average correlation between \"none\" network and retrosplenial temporal network | rsfMRI_rsfmri_c_ngd_cgc_ngd_rspltp | **6** | 0.0448 |
| **rsfMRI** | Average correlation between cingulo-parietal network and salience network | rsfMRI_rsfmri_c_ngd_ca_ngd_sa | **7** | 0.0444 |
| **sMRI** | Cortical volume in APARC ROI lh-caudalanteriorcingulate | sMRI_smri_vol_cdk_cdacatelh | **8** | 0.0442 |
| **rsfMRI** | Average correlation between default network and dorsal attention network | rsfMRI_rsfmri_c_ngd_dt_ngd_dla | **9** | 0.0438 |
| **rsfMRI** | Average correlation between fronto-parietal network and fronto-parietal network | rsfMRI_rsfmri_c_ngd_fo_ngd_fo | **10** | 0.0416 |
| **rsfMRI** | Average correlation between cingulo-opercular network and cingulo-parietal network | rsfMRI_rsfmri_c_ngd_cgc_ngd_ca | **11** | 0.0415 |
| **rsfMRI** | Average correlation between auditory network and cingulo-parietal network | rsfMRI_rsfmri_c_ngd_ad_ngd_ca | **12** | 0.0390 |
| **sMRI** | Cortical area in APARC ROI lh-caudalanteriorcingulate | sMRI_smri_area_cdk_cdacatelh | **13** | 0.0346 |
| **rsfMRI** | Average correlation between fronto-parietal network and ventral attention network | rsfMRI_rsfmri_c_ngd_fo_ngd_vta | **14** | 0.0331 |
| **sMRI** | Cortical volume in APARC ROI rh-cuneus | sMRI_smri_vol_cdk_cuneusrh | **15** | 0.0324 |
| **rsfMRI** | Average correlation between auditory network and ventral attention network | rsfMRI_rsfmri_c_ngd_ad_ngd_vta | **16** | 0.0320 |
| **rsfMRI** | Average correlation between dorsal attention network and ventral attention network | rsfMRI_rsfmri_c_ngd_dla_ngd_vta | **17** | 0.0313 |
| **rsfMRI** | Average correlation between cingulo-opercular network and default network | rsfMRI_rsfmri_c_ngd_cgc_ngd_dt | **18** | 0.0310 |
| **rsfMRI** | Average correlation between default network and fronto-parietal network | rsfMRI_rsfmri_c_ngd_dt_ngd_fo | **19** | 0.0309 |
| **DTI** | Fiber tract volume in DTI atlas tract left parahippocampal cingulum | DTI_dmdtifp1_174 | **20** | 0.0305 |
| **DTI** | Fiber tract volume in DTI atlas tract left fornix, excluding fimbria | DTI_dmdtifp1_205 | **21** | 0.0304 |
| **rsfMRI** | Average correlation between retrosplenial temporal network and retrosplenial temporal | rsfMRI_rsfmri_c_ngd_rspltp_ngd_rspltp | **22** | 0.0298 |
| **sMRI** | Cortical volume in APARC ROI rh-entorhinal | sMRI_smri_vol_cdk_ehinalrh | **23** | 0.0284 |
| **sMRI** | Cortical area in APARC ROI rh-entorhinal | sMRI_smri_area_cdk_ehinalrh | **24** | 0.0281 |
| **sMRI** | Cortical area in APARC ROI lh-frontalpole | sMRI_smri_area_cdk_frpolelh | **25** | 0.0234 |
| **rsfMRI** | Average correlation between cingulo-opercular network and \"none\" network | rsfMRI_rsfmri_c_ngd_cgc_ngd_n | **26** | 0.0205 |
| **rsfMRI** | Average correlation between default network and ventral attention network | rsfMRI_rsfmri_c_ngd_dt_ngd_vta | **27** | 0.0189 |
| **RSI** | Neurite density within DTI atlas tract right temporal superior longitudinal fasiculus | RSI_dmri_rsind_fiberat_tslfrh | **28** | 0.0168 |

**3. Multimodal Multiple Logistic Regression**

Results from our multiple regression model with multiple comparison correction (adjusted p-value) are listed in the following:

**3.1. structural MRI**

|  | thickness | | | | sulcal depth | | | |
| --- | --- | --- | --- | --- | --- | --- | --- | --- |
| dict | Coefficient | 95% CI | | Adjusted p-value | Coefficient | 95% CI | | Adjusted p-value |
| lh-Banks of Superior Temporal Sulcus | 0.272158 | -0.05807 | 0.60277 | 0.460853 | 0.426425 | -0.01386 | 0.870816 | 0.130437 |
| lh-caudalanteriorcingulate | -0.13849 | -0.37179 | 0.094207 | 0.641586 | 1.970154 | 0.852448 | 3.089556 | 0.004927 |
| lh-caudalmiddlefrontal | -0.10381 | -0.48282 | 0.276209 | 0.724454 | 1.233355 | 0.207531 | 2.255194 | 0.064607 |
| lh-cuneus | 0.21319 | -0.15616 | 0.582339 | 0.645278 | -0.87515 | -1.66507 | -0.08599 | 0.094527 |
| lh-entorhinal | -0.0815 | -0.25383 | 0.090723 | 0.657102 | -0.26683 | -0.73485 | 0.199868 | 0.366239 |
| lh-fusiform | 0.317894 | -0.14068 | 0.777175 | 0.535036 | -0.29832 | -1.39312 | 0.795456 | 0.67916 |
| lh-inferiorparietal | 0.123456 | -0.24649 | 0.495726 | 0.657102 | 1.245254 | -0.13995 | 2.630097 | 0.158226 |
| lh-inferiortemporal | 0.125136 | -0.23104 | 0.482073 | 0.657102 | 1.094146 | 0.029471 | 2.158795 | 0.114432 |
| lh-isthmuscingulate | 0.435258 | 0.140478 | 0.729574 | 0.133877 | -0.04184 | -1.13428 | 1.050909 | 0.953593 |
| lh-lateraloccipital | -0.06897 | -0.43367 | 0.296355 | 0.788864 | 0.029371 | -1.11669 | 1.173821 | 0.959905 |
| lh-lateralorbitofrontal | 0.19517 | -0.17761 | 0.568236 | 0.645278 | 0.133531 | -1.35564 | 1.623604 | 0.929344 |
| lh-lingual | -0.04655 | -0.47261 | 0.379169 | 0.879928 | 0.425489 | -0.74555 | 1.593987 | 0.603293 |
| lh-medialorbitofrontal | 0.109382 | -0.21986 | 0.438508 | 0.657102 | -1.46556 | -2.52872 | -0.40257 | 0.034913 |
| lh-middletemporal | 0.285559 | -0.01378 | 0.587485 | 0.331035 | -0.97312 | -2.00423 | 0.053964 | 0.137258 |
| lh-parahippocampal | -0.10921 | -0.31076 | 0.092271 | 0.645278 | 0.438125 | -0.19302 | 1.06709 | 0.285384 |
| lh-paracentral | -0.22195 | -0.58329 | 0.139151 | 0.623781 | 0.729084 | -0.47611 | 1.934342 | 0.348633 |
| lh-parsopercularis | 0.302945 | -0.08055 | 0.687613 | 0.465552 | 0.667791 | -0.37542 | 1.707586 | 0.322267 |
| lh-parsorbitalis | 0.450575 | 0.178694 | 0.722914 | 0.083143 | -1.07421 | -1.94887 | -0.20172 | 0.059543 |
| lh-parstriangularis | 0.149241 | -0.20943 | 0.508681 | 0.657102 | -0.30392 | -1.23973 | 0.629316 | 0.633697 |
| lh-pericalcarine | -0.12685 | -0.50569 | 0.250889 | 0.657102 | -0.60139 | -1.27973 | 0.079808 | 0.163432 |
| lh-postcentral | -0.12474 | -0.48599 | 0.236016 | 0.657102 | -0.55876 | -2.302 | 1.157517 | 0.633697 |
| lh-posteriorcingulate | 0.242661 | -0.1132 | 0.597764 | 0.535036 | 3.305969 | 1.977167 | 4.637432 | 2.61E-05 |
| lh-precentral | -0.12903 | -0.51079 | 0.254741 | 0.657102 | -1.27667 | -3.2667 | 0.711526 | 0.322267 |
| lh-precuneus | 0.169331 | -0.27211 | 0.611349 | 0.657102 | -1.60214 | -2.88131 | -0.32441 | 0.055333 |
| lh-rostralanteriorcingulate | 0.221015 | -0.05032 | 0.492369 | 0.460853 | 1.753877 | 0.772553 | 2.737035 | 0.004716 |
| lh-rostralmiddlefrontal | 0.207707 | -0.16995 | 0.587563 | 0.645278 | -1.55019 | -3.69937 | 0.594972 | 0.278684 |
| lh-superiorfrontal | 0.167995 | -0.19685 | 0.533655 | 0.657102 | -2.80488 | -5.32655 | -0.28421 | 0.094527 |
| lh-superiorparietal | 0.317183 | -0.09384 | 0.729878 | 0.465552 | 1.459861 | 0.012938 | 2.908888 | 0.114432 |
| lh-superiortemporal | 0.373762 | 0.053224 | 0.695474 | 0.22838 | -1.91657 | -3.3912 | -0.44773 | 0.044642 |
| lh-supramarginal | 0.028122 | -0.29919 | 0.357657 | 0.895342 | 0.100618 | -1.318 | 1.515792 | 0.929344 |
| lh-frontalpole | 0.229294 | 0.051785 | 0.407231 | 0.162429 | -1.0569 | -1.59008 | -0.52607 | 0.001399 |
| lh-temporalpole | 0.186807 | -0.01148 | 0.385818 | 0.331035 | -0.88372 | -1.51301 | -0.25602 | 0.034595 |
| lh-transversetemporal | 0.356583 | 0.086363 | 0.62686 | 0.162429 | 1.266077 | 0.617221 | 1.915262 | 0.001554 |
| lh-insula | 0.050922 | -0.35288 | 0.455128 | 0.865824 | 2.458004 | 1.4868 | 3.434108 | 2.61E-05 |
| rh-Banks of Superior Temporal Sulcus | 0.12506 | -0.18368 | 0.433908 | 0.657102 | 0.611274 | 0.006834 | 1.220714 | 0.114432 |
| rh-caudalanteriorcingulate | 0.113263 | -0.15778 | 0.383438 | 0.657102 | 1.167293 | 0.015898 | 2.320175 | 0.114432 |
| rh-caudalmiddlefrontal | -0.00018 | -0.36956 | 0.370789 | 0.999241 | 1.138612 | 0.319653 | 1.956051 | 0.034609 |
| rh-cuneus | 0.090604 | -0.26631 | 0.447725 | 0.732313 | -0.48659 | -1.27819 | 0.302842 | 0.343807 |
| rh-entorhinal | -0.03644 | -0.18784 | 0.115373 | 0.73819 | -0.52293 | -1.00277 | -0.04443 | 0.095892 |
| rh-fusiform | -0.03614 | -0.4692 | 0.397427 | 0.895342 | 0.807276 | -0.33494 | 1.949576 | 0.284652 |
| rh-inferiorparietal | 0.157806 | -0.21861 | 0.536693 | 0.657102 | 0.805396 | -0.68119 | 2.290855 | 0.393301 |
| rh-inferiortemporal | 0.073904 | -0.28504 | 0.433759 | 0.77411 | -0.61222 | -1.68266 | 0.45488 | 0.366239 |
| rh-isthmuscingulate | 0.259239 | -0.03353 | 0.551123 | 0.388805 | 0.491111 | -0.49357 | 1.474075 | 0.439072 |
| rh-lateraloccipital | -0.11178 | -0.46797 | 0.245095 | 0.671166 | -0.7109 | -1.95618 | 0.530869 | 0.366239 |
| rh-lateralorbitofrontal | 0.127424 | -0.23061 | 0.485733 | 0.657102 | 0.519392 | -0.85281 | 1.8935 | 0.593227 |
| rh-lingual | 0.063971 | -0.35776 | 0.485404 | 0.836848 | 1.368409 | 0.128032 | 2.609504 | 0.094527 |
| rh-medialorbitofrontal | 0.339369 | 0.019731 | 0.659324 | 0.250403 | -3.53104 | -4.89903 | -2.16439 | 2.61E-05 |
| rh-middletemporal | 0.320043 | 0.015301 | 0.627314 | 0.250403 | -0.15086 | -1.41466 | 1.110581 | 0.912268 |
| rh-parahippocampal | 0.106527 | -0.12981 | 0.343074 | 0.657102 | 0.086044 | -0.66575 | 0.836488 | 0.912268 |
| rh-paracentral | -0.21029 | -0.61555 | 0.194847 | 0.645278 | 0.3424 | -0.6886 | 1.374252 | 0.633697 |
| rh-parsopercularis | 0.405463 | 0.037419 | 0.774137 | 0.250403 | 0.806125 | 0.030508 | 1.579527 | 0.112832 |
| rh-parsorbitalis | 0.33065 | 0.058571 | 0.603165 | 0.204706 | -0.8552 | -1.78711 | 0.074008 | 0.149576 |
| rh-parstriangularis | 0.361134 | 0.015598 | 0.708326 | 0.250403 | -0.31444 | -1.31106 | 0.678518 | 0.633697 |
| rh-pericalcarine | -0.1236 | -0.49907 | 0.251017 | 0.657102 | -0.50288 | -1.14773 | 0.145704 | 0.238038 |
| rh-postcentral | -0.0893 | -0.43028 | 0.250713 | 0.730677 | -1.16766 | -2.64836 | 0.29192 | 0.229293 |
| rh-posteriorcingulate | 0.163957 | -0.24381 | 0.570083 | 0.657102 | 2.136199 | 0.839361 | 3.436159 | 0.008129 |
| rh-precentral | -0.19693 | -0.5848 | 0.192947 | 0.65085 | -0.13059 | -1.90392 | 1.639548 | 0.929344 |
| rh-precuneus | 0.228175 | -0.20481 | 0.662799 | 0.645278 | -1.23043 | -2.38841 | -0.07224 | 0.105866 |
| rh-rostralanteriorcingulate | 0.201155 | -0.05689 | 0.459097 | 0.465552 | 1.580182 | 0.632491 | 2.528633 | 0.007712 |
| rh-rostralmiddlefrontal | 0.394166 | 0.014792 | 0.775902 | 0.250403 | -1.30858 | -3.29589 | 0.675995 | 0.317053 |
| rh-superiorfrontal | 0.263096 | -0.13144 | 0.658486 | 0.544235 | -3.09541 | -5.44146 | -0.75098 | 0.042932 |
| rh-superiorparietal | 0.217987 | -0.19836 | 0.635908 | 0.645278 | 0.062839 | -1.29877 | 1.424009 | 0.953593 |
| rh-superiortemporal | 0.480502 | 0.134264 | 0.827532 | 0.155795 | -2.32492 | -4.03563 | -0.61653 | 0.036352 |
| rh-supramarginal | 0.073109 | -0.23654 | 0.38487 | 0.73819 | 0.848687 | -0.29192 | 1.988174 | 0.26305 |
| rh-frontalpole | 0.068862 | -0.11682 | 0.254917 | 0.657102 | -0.1638 | -0.72741 | 0.398135 | 0.661487 |
| rh-temporalpole | 0.137808 | -0.05163 | 0.328301 | 0.500261 | -0.59874 | -1.21474 | 0.015352 | 0.129067 |
| rh-transversetemporal | 0.204352 | -0.07588 | 0.484727 | 0.500261 | 1.112533 | 0.457775 | 1.768416 | 0.006904 |
| rh-insula | -0.02379 | -0.39017 | 0.342931 | 0.911589 | 2.193304 | 1.184628 | 3.204926 | 0.000369 |
| Mean left hemisphere | 0.220451 | -0.30902 | 0.751328 | 0.657102 | 22.4431 | -9.51354 | 54.36573 | 0.284652 |
| Mean right hemisphere | 0.264806 | -0.2822 | 0.813035 | 0.657102 | -2.27084 | -34.4933 | 29.92185 | 0.929344 |
| Mean whole brain | 0.248371 | -0.29683 | 0.79488 | 0.657102 | 14.42985 | -23.8226 | 52.66667 | 0.593227 |

|  | Cortical surface area | | | | volume | | | |
| --- | --- | --- | --- | --- | --- | --- | --- | --- |
| dict | Coefficient | 95% CI | | Adjusted p-value | Coefficient | 95% CI | | Adjusted p-value |
| lh-Banks of Superior Temporal Sulcus | -0.0004 | -0.00069 | -0.00012 | 0.00634 | -0.00011 | -0.00021 | -2.04E-05 | 0.022755 |
| lh-caudalanteriorcingulate | -0.00084 | -0.0012 | -0.00049 | 6.77E-06 | -0.00022 | -0.00031 | -0.00013 | 1.04E-05 |
| lh-caudalmiddlefrontal | -0.00023 | -0.00035 | -0.0001 | 0.000518 | -6.35E-05 | -9.99E-05 | -2.71E-05 | 0.001049 |
| lh-cuneus | -0.00036 | -0.00063 | -9.43E-05 | 0.009351 | -5.47E-05 | -0.00015 | 4.06E-05 | 0.268774 |
| lh-entorhinal | -0.00086 | -0.00155 | -0.00018 | 0.015049 | -0.00016 | -0.00031 | -1.89E-05 | 0.033501 |
| lh-fusiform | -0.0003 | -0.00043 | -0.00016 | 2.23E-05 | -6.00E-05 | -9.49E-05 | -2.53E-05 | 0.001129 |
| lh-inferiorparietal | -0.00019 | -0.00026 | -0.00011 | 6.03E-06 | -5.80E-05 | -8.29E-05 | -3.32E-05 | 1.80E-05 |
| lh-inferiortemporal | -0.00029 | -0.0004 | -0.00017 | 1.73E-06 | -7.50E-05 | -0.0001 | -4.61E-05 | 2.45E-06 |
| lh-isthmuscingulate | -0.00071 | -0.001 | -0.00043 | 2.74E-06 | -0.00019 | -0.00028 | -9.73E-05 | 0.000142 |
| lh-lateraloccipital | -0.00018 | -0.00027 | -9.25E-05 | 0.00011 | -6.76E-05 | -9.92E-05 | -3.62E-05 | 5.82E-05 |
| lh-lateralorbitofrontal | -0.00052 | -0.00072 | -0.00032 | 8.41E-07 | -0.00013 | -0.00019 | -7.64E-05 | 2.01E-05 |
| lh-lingual | -0.00019 | -0.00032 | -5.89E-05 | 0.005686 | -4.94E-05 | -9.61E-05 | -2.75E-06 | 0.044781 |
| lh-medialorbitofrontal | -0.00056 | -0.00079 | -0.00033 | 4.29E-06 | -0.00013 | -0.00019 | -6.03E-05 | 0.000356 |
| lh-middletemporal | -0.00032 | -0.00045 | -0.00019 | 2.74E-06 | -6.46E-05 | -9.68E-05 | -3.25E-05 | 0.00016 |
| lh-parahippocampal | -0.0003 | -0.00073 | 0.000119 | 0.169267 | -9.29E-05 | -0.00022 | 3.34E-05 | 0.165091 |
| lh-paracentral | -0.00041 | -0.00068 | -0.00015 | 0.003133 | -0.00013 | -0.00021 | -5.16E-05 | 0.001723 |
| lh-parsopercularis | -0.0004 | -0.00059 | -0.00022 | 3.00E-05 | -9.56E-05 | -0.00015 | -4.30E-05 | 0.000655 |
| lh-parsorbitalis | -0.00137 | -0.00204 | -0.00071 | 7.54E-05 | -0.00017 | -0.00032 | -2.18E-05 | 0.031664 |
| lh-parstriangularis | -0.00045 | -0.0007 | -0.00019 | 0.000925 | -9.83E-05 | -0.00017 | -2.60E-05 | 0.010687 |
| lh-pericalcarine | -0.00026 | -0.0005 | -2.95E-05 | 0.030317 | -0.00015 | -0.00027 | -1.97E-05 | 0.030609 |
| lh-postcentral | -0.00026 | -0.00036 | -0.00016 | 1.95E-06 | -8.28E-05 | -0.00012 | -4.99E-05 | 4.29E-06 |
| lh-posteriorcingulate | -0.00069 | -0.00096 | -0.00042 | 2.04E-06 | -0.00019 | -0.00027 | -0.0001 | 3.56E-05 |
| lh-precentral | -0.00022 | -0.00032 | -0.00012 | 1.91E-05 | -7.66E-05 | -0.00011 | -4.51E-05 | 8.78E-06 |
| lh-precuneus | -0.00032 | -0.00043 | -0.00021 | 1.98E-07 | -8.49E-05 | -0.00012 | -4.80E-05 | 2.17E-05 |
| lh-rostralanteriorcingulate | -0.00096 | -0.00132 | -0.0006 | 8.39E-07 | -0.00024 | -0.00034 | -0.00014 | 4.34E-06 |
| lh-rostralmiddlefrontal | -0.00022 | -0.00029 | -0.00015 | 1.48E-08 | -6.82E-05 | -9.11E-05 | -4.55E-05 | 8.04E-08 |
| lh-superiorfrontal | -0.0002 | -0.00026 | -0.00013 | 1.48E-08 | -5.26E-05 | -7.14E-05 | -3.39E-05 | 5.11E-07 |
| lh-superiorparietal | -0.00013 | -0.00021 | -5.48E-05 | 0.001083 | -3.85E-05 | -6.51E-05 | -1.19E-05 | 0.006334 |
| lh-superiortemporal | -0.00035 | -0.00047 | -0.00023 | 4.71E-08 | -6.54E-05 | -9.78E-05 | -3.32E-05 | 0.000143 |
| lh-supramarginal | -0.0002 | -0.00029 | -0.00011 | 1.78E-05 | -6.24E-05 | -9.00E-05 | -3.49E-05 | 2.63E-05 |
| lh-frontalpole | -0.00359 | -0.00511 | -0.00207 | 8.63E-06 | -0.00033 | -0.00063 | -2.06E-05 | 0.044218 |
| lh-temporalpole | -0.00155 | -0.00247 | -0.00064 | 0.001144 | -0.00012 | -0.00027 | 3.46E-05 | 0.142355 |
| lh-transversetemporal | -0.00167 | -0.00239 | -0.00095 | 1.00E-05 | -0.00031 | -0.00052 | -0.00011 | 0.003683 |
| lh-insula | -0.00064 | -0.00088 | -0.00041 | 6.10E-07 | -0.00018 | -0.00025 | -0.00011 | 2.74E-06 |
| rh-Banks of Superior Temporal Sulcus | -0.00077 | -0.00111 | -0.00043 | 1.78E-05 | -0.00018 | -0.00029 | -7.49E-05 | 0.001464 |
| rh-caudalanteriorcingulate | -0.00077 | -0.0011 | -0.00045 | 7.67E-06 | -0.00019 | -0.00028 | -9.95E-05 | 6.85E-05 |
| rh-caudalmiddlefrontal | -0.00028 | -0.0004 | -0.00016 | 9.55E-06 | -8.05E-05 | -0.00012 | -4.47E-05 | 3.11E-05 |
| rh-cuneus | -0.00036 | -0.0006 | -0.00012 | 0.004031 | -7.98E-05 | -0.00016 | 3.84E-06 | 0.069996 |
| rh-entorhinal | -9.04E-05 | -0.00071 | 0.000521 | 0.772652 | -3.06E-05 | -0.00017 | 0.000107 | 0.664694 |
| rh-fusiform | -0.00037 | -0.00051 | -0.00024 | 4.09E-07 | -9.14E-05 | -0.00013 | -5.50E-05 | 4.29E-06 |
| rh-inferiorparietal | -0.00015 | -0.00022 | -7.45E-05 | 8.64E-05 | -4.13E-05 | -6.35E-05 | -1.92E-05 | 0.000445 |
| rh-inferiortemporal | -0.00038 | -0.00051 | -0.00026 | 1.48E-08 | -8.51E-05 | -0.00012 | -5.44E-05 | 6.35E-07 |
| rh-isthmuscingulate | -0.0003 | -0.00061 | 8.67E-06 | 0.062058 | -6.27E-05 | -0.00016 | 3.25E-05 | 0.21068 |
| rh-lateraloccipital | -0.00024 | -0.00033 | -0.00015 | 8.39E-07 | -7.91E-05 | -0.00011 | -4.93E-05 | 1.44E-06 |
| rh-lateralorbitofrontal | -0.00053 | -0.00071 | -0.00035 | 1.08E-07 | -0.00015 | -0.0002 | -9.24E-05 | 1.44E-06 |
| rh-lingual | -0.00015 | -0.00029 | -1.70E-05 | 0.030317 | -3.97E-05 | -8.84E-05 | 8.79E-06 | 0.120961 |
| rh-medialorbitofrontal | -0.00077 | -0.00105 | -0.0005 | 1.87E-07 | -0.00015 | -0.00022 | -6.89E-05 | 0.000356 |
| rh-middletemporal | -0.00041 | -0.00053 | -0.00028 | 1.57E-09 | -8.70E-05 | -0.00012 | -5.55E-05 | 6.35E-07 |
| rh-parahippocampal | -0.00046 | -0.00089 | -3.90E-05 | 0.0375 | -8.31E-05 | -0.00021 | 4.43E-05 | 0.216967 |
| rh-paracentral | -0.00049 | -0.00071 | -0.00026 | 3.77E-05 | -0.00015 | -0.00022 | -8.00E-05 | 4.82E-05 |
| rh-parsopercularis | -0.00044 | -0.00065 | -0.00023 | 4.91E-05 | -0.0001 | -0.00016 | -3.76E-05 | 0.002603 |
| rh-parsorbitalis | -0.00141 | -0.00195 | -0.00087 | 1.03E-06 | -0.00028 | -0.0004 | -0.00015 | 3.94E-05 |

**3.2. Diffusion MRI**

|  | axial diffusivity | | | | fractional anisotropy | | | |
| --- | --- | --- | --- | --- | --- | --- | --- | --- |
| dict | Coefficient | 95% CI | | Adjusted p-value | Coefficient | 95% CI | | Adjusted p-value |
| right fornix | 0.866978 | -0.25698 | 1.980216 | 0.45784 | -1.11338 | -3.2501 | 1.026065 | 0.322724 |
| left fornix | 0.797939 | -0.38454 | 1.96968 | 0.45784 | -1.34178 | -3.52054 | 0.841959 | 0.258671 |
| right cingulate cingulum | 0.877976 | -0.45633 | 2.207041 | 0.45784 | -1.024 | -2.34041 | 0.294457 | 0.157618 |
| left cingulate cingulum | 0.59011 | -0.60811 | 1.78429 | 0.546453 | -0.91483 | -2.20177 | 0.376318 | 0.196989 |
| right parahippocampal cingulum | 0.820415 | -0.42857 | 2.059742 | 0.45784 | -1.48969 | -3.21675 | 0.241444 | 0.119759 |
| left parahippocampal cingulum | 0.894225 | -0.25949 | 2.039935 | 0.45784 | -1.28783 | -2.91308 | 0.34232 | 0.153879 |
| right corticospinal/pyramidal | 0.705426 | -0.77589 | 2.177082 | 0.546453 | -3.69885 | -5.9088 | -1.49783 | 0.002625 |
| left corticospinal/pyramidal | 0.852377 | -0.66397 | 2.359071 | 0.537925 | -3.8193 | -5.97787 | -1.67026 | 0.001626 |
| right anterior thalamic radiations | 1.552528 | -0.02319 | 3.117329 | 0.368199 | -1.28809 | -3.48279 | 0.908935 | 0.269439 |
| left anterior thalamic radiations | 1.483731 | -0.07747 | 3.034444 | 0.369466 | -1.344 | -3.45976 | 0.775287 | 0.248999 |
| right uncinate | 2.279751 | 0.472873 | 4.082728 | 0.186034 | -2.64979 | -4.74524 | -0.54715 | 0.021542 |
| left uncinate | 1.712365 | 0.076383 | 3.343187 | 0.334953 | -1.10631 | -2.97514 | 0.769884 | 0.269439 |
| right inferior longitudinal fasiculus | 0.558505 | -1.04754 | 2.15588 | 0.669651 | -2.38215 | -4.48063 | -0.27885 | 0.03935 |
| left inferior longitudinal fasiculus | 0.1379 | -1.52894 | 1.797923 | 0.914453 | -3.29832 | -5.35233 | -1.24025 | 0.003671 |
| right inferior-fronto-occipital fasiculus | 0.729607 | -1.17666 | 2.626872 | 0.654646 | -3.58734 | -5.85123 | -1.31567 | 0.004048 |
| left inferior-fronto-occipital fasiculus | 0.953577 | -0.88871 | 2.78888 | 0.546453 | -3.05448 | -5.20109 | -0.90077 | 0.009775 |
| foreceps major | 1.705514 | 0.257838 | 3.15841 | 0.222298 | -0.57211 | -2.04207 | 0.932315 | 0.461452 |
| foreceps minor | 0.505252 | -0.87375 | 1.883397 | 0.661492 | -2.51607 | -4.14877 | -0.87429 | 0.005177 |
| corpus callosum | 0.969733 | -0.5641 | 2.498442 | 0.47406 | -3.07618 | -5.32807 | -0.81463 | 0.012646 |
| right superior longitudinal fasiculus | 0.509713 | -1.97502 | 2.986775 | 0.848813 | -3.72076 | -5.91716 | -1.51831 | 0.002552 |
| left superior longitudinal fasiculus | 1.174819 | -1.17007 | 3.513111 | 0.546453 | -3.7434 | -5.867 | -1.61214 | 0.001683 |
| right temporal superior longitudinal fasiculus | 0.755179 | -1.61479 | 3.118458 | 0.697775 | -2.06088 | -4.19265 | 0.078556 | 0.079261 |
| left temporal superior longitudinal fasiculus | 1.103308 | -1.09708 | 3.29741 | 0.546453 | -2.94479 | -5.00627 | -0.8744 | 0.009775 |
| right parietal superior longitudinal fasiculus | 0.536938 | -1.95549 | 3.022308 | 0.848813 | -4.18952 | -6.36538 | -2.00804 | 0.000686 |
| left parietal superior longitudinal fasiculus | 1.476305 | -1.03808 | 3.98509 | 0.523369 | -4.58091 | -6.70663 | -2.44954 | 0.000225 |
| right superior corticostriate | -0.10717 | -1.84314 | 1.617128 | 0.925383 | -3.06745 | -4.9339 | -1.20629 | 0.002927 |
| left superior corticostriate | 0.192568 | -1.83961 | 2.211247 | 0.914453 | -5.31393 | -7.65636 | -2.97802 | 0.000194 |
| right superior corticostriate-frontal cortex only | 0.245982 | -1.52596 | 2.005599 | 0.867401 | -2.58635 | -4.4733 | -0.70499 | 0.012462 |
| left superior corticostriate-frontal cortex only | 0.297073 | -1.74389 | 2.323731 | 0.867401 | -4.74818 | -7.00096 | -2.50193 | 0.000245 |
| right superior corticostriate-parietal cortex only | -0.26059 | -1.9527 | 1.421475 | 0.867401 | -2.99988 | -4.81108 | -1.19246 | 0.002849 |
| left superior corticostriate-parietal cortex only | -0.07571 | -1.92885 | 1.76675 | 0.935984 | -4.36837 | -6.41008 | -2.33145 | 0.000225 |
| right striatal inferior frontal cortex | 3.32538 | 0.955585 | 5.692123 | 0.124198 | -2.39575 | -4.39608 | -0.39065 | 0.029598 |
| left striatal inferior frontal cortex | 4.278265 | 1.85612 | 6.699739 | 0.022446 | -2.25934 | -4.358 | -0.15243 | 0.049252 |
| right inferior frontal superior frontal cortex | -0.37648 | -2.80457 | 2.044163 | 0.867401 | -4.92331 | -7.33931 | -2.50593 | 0.000341 |
| left inferior frontal superior frontal cortex | 0.907429 | -1.42301 | 3.231814 | 0.654646 | -5.15237 | -7.47534 | -2.82726 | 0.000194 |
| right fornix, excluding fimbria | 0.756062 | -0.2832 | 1.786227 | 0.45784 | 0.526071 | -1.3085 | 2.357593 | 0.573721 |
| left fornix, excluding fimbria | 0.551652 | -0.61289 | 1.707524 | 0.546453 | -2.23771 | -4.29998 | -0.17863 | 0.048205 |
| all DTI atlas tract fibers | 1.449288 | -0.49797 | 3.387222 | 0.45784 | -5.75871 | -8.77619 | -2.73327 | 0.000711 |
| right hemisphere DTI atlas tract fibers without corpus callosum | 1.497185 | -0.6955 | 3.677767 | 0.45784 | -6.75461 | -10.0122 | -3.49252 | 0.000291 |
| left hemisphere DTI atlas tract fibers without corpus callosum | 1.561007 | -0.59096 | 3.701338 | 0.45784 | -7.00788 | -10.123 | -3.88936 | 0.000194 |
| right hemisphere DTI atlas tract fibers | 1.389282 | -0.56872 | 3.337805 | 0.45784 | -5.51927 | -8.54694 | -2.48308 | 0.001253 |
| left hemisphere DTI atlas tract fibers | 1.466496 | -0.42635 | 3.350595 | 0.45784 | -5.62897 | -8.54059 | -2.7098 | 0.000686 |

|  | fiber tract | | | | mean diffusivity | | | |
| --- | --- | --- | --- | --- | --- | --- | --- | --- |
| dict | Coefficient | 95% CI | | Adjusted p-value | Coefficient | 95% CI | | Adjusted p-value |
| right fornix | -0.00018 | -0.00026 | -9.29E-05 | 5.26E-05 | 2.054884 | 0.233042 | 3.861161 | 0.029151 |
| left fornix | -0.00019 | -0.00027 | -0.0001 | 2.03E-05 | 1.960886 | 0.08225 | 3.824815 | 0.04195 |
| right cingulate cingulum | -0.00022 | -0.00032 | -0.00012 | 1.05E-05 | 2.929245 | 0.738489 | 5.113787 | 0.012995 |
| left cingulate cingulum | -0.00018 | -0.00027 | -8.46E-05 | 0.000216 | 2.882866 | 0.507802 | 5.250549 | 0.020912 |
| right parahippocampal cingulum | -0.00023 | -0.00038 | -9.02E-05 | 0.0016 | 2.117719 | 0.331548 | 3.891355 | 0.022959 |
| left parahippocampal cingulum | -0.00011 | -0.00026 | 4.93E-05 | 0.178553 | 2.21334 | 0.456671 | 3.958306 | 0.017331 |
| right corticospinal/pyramidal | -0.00016 | -0.00022 | -9.54E-05 | 1.27E-06 | 4.277864 | 1.44533 | 7.090016 | 0.006157 |
| left corticospinal/pyramidal | -0.00014 | -0.0002 | -8.49E-05 | 4.26E-06 | 4.834394 | 1.96958 | 7.679849 | 0.003158 |
| right anterior thalamic radiations | -9.56E-05 | -0.00014 | -5.61E-05 | 4.60E-06 | 4.43313 | 1.635764 | 7.217247 | 0.004307 |
| left anterior thalamic radiations | -8.92E-05 | -0.00013 | -5.15E-05 | 7.12E-06 | 4.451467 | 1.655216 | 7.234486 | 0.004307 |
| right uncinate | -0.00011 | -0.00017 | -5.57E-05 | 0.000107 | 4.404189 | 2.038502 | 6.760851 | 0.001343 |
| left uncinate | -0.0001 | -0.00018 | -2.95E-05 | 0.006178 | 3.645313 | 1.130645 | 6.150478 | 0.007649 |
| right inferior longitudinal fasiculus | -8.12E-05 | -0.00011 | -4.86E-05 | 2.61E-06 | 2.937559 | 0.290582 | 5.575067 | 0.031558 |
| left inferior longitudinal fasiculus | -7.21E-05 | -0.0001 | -4.06E-05 | 1.25E-05 | 3.259464 | 0.568131 | 5.942883 | 0.020912 |
| right inferior-fronto-occipital fasiculus | -8.29E-05 | -0.00012 | -5.01E-05 | 2.12E-06 | 3.509901 | 0.869922 | 6.136109 | 0.012995 |
| left inferior-fronto-occipital fasiculus | -7.75E-05 | -0.00011 | -4.19E-05 | 2.91E-05 | 4.049173 | 1.310787 | 6.776105 | 0.007018 |
| foreceps major | -3.07E-05 | -5.34E-05 | -7.90E-06 | 0.008445 | 2.527235 | 0.468059 | 4.573881 | 0.020122 |
| foreceps minor | -4.14E-05 | -5.92E-05 | -2.37E-05 | 9.14E-06 | 2.566105 | 0.614764 | 4.506726 | 0.013632 |
| corpus callosum | -1.19E-05 | -1.69E-05 | -6.97E-06 | 4.93E-06 | 3.119133 | 0.734162 | 5.49204 | 0.013769 |
| right superior longitudinal fasiculus | -8.14E-05 | -0.00012 | -4.56E-05 | 1.42E-05 | 4.650737 | 1.312589 | 7.973701 | 0.009999 |
| left superior longitudinal fasiculus | -7.45E-05 | -0.00011 | -3.65E-05 | 0.00015 | 5.324268 | 2.114907 | 8.521464 | 0.003618 |
| right temporal superior longitudinal fasiculus | -9.19E-05 | -0.00014 | -4.44E-05 | 0.000184 | 3.207107 | -0.07235 | 6.469165 | 0.055883 |
| left temporal superior longitudinal fasiculus | -8.49E-05 | -0.00013 | -4.03E-05 | 0.00022 | 4.579371 | 1.408437 | 7.737242 | 0.007649 |
| right parietal superior longitudinal fasiculus | -9.36E-05 | -0.00013 | -5.63E-05 | 2.40E-06 | 5.302896 | 1.957744 | 8.634149 | 0.004307 |
| left parietal superior longitudinal fasiculus | -8.44E-05 | -0.00012 | -4.44E-05 | 5.04E-05 | 6.578789 | 3.336943 | 9.810441 | 0.000995 |
| right superior corticostriate | -9.08E-05 | -0.00013 | -5.61E-05 | 1.27E-06 | 6.151505 | 2.322394 | 9.964285 | 0.004307 |
| left superior corticostriate | -5.69E-05 | -7.73E-05 | -3.66E-05 | 2.27E-07 | 7.516206 | 3.717317 | 11.30126 | 0.000995 |
| right superior corticostriate-frontal cortex only | -8.20E-05 | -0.00011 | -5.05E-05 | 1.27E-06 | 5.818708 | 1.956399 | 9.664796 | 0.006157 |
| left superior corticostriate-frontal cortex only | -6.67E-05 | -9.09E-05 | -4.27E-05 | 2.71E-07 | 7.493912 | 3.670253 | 11.30341 | 0.000995 |
| right superior corticostriate-parietal cortex only | -0.00012 | -0.00016 | -7.05E-05 | 2.10E-06 | 5.311693 | 1.67664 | 8.930646 | 0.007472 |
| left superior corticostriate-parietal cortex only | -0.0001 | -0.00014 | -6.40E-05 | 1.27E-06 | 6.414355 | 2.86709 | 9.946863 | 0.001782 |
| right striatal inferior frontal cortex | -9.31E-05 | -0.00014 | -5.00E-05 | 3.46E-05 | 5.392214 | 2.655605 | 8.116027 | 0.000995 |
| left striatal inferior frontal cortex | -8.55E-05 | -0.00013 | -4.37E-05 | 8.09E-05 | 6.294863 | 3.396303 | 9.182159 | 0.000837 |
| right inferior frontal superior frontal cortex | -7.65E-05 | -0.0001 | -5.24E-05 | 2.60E-08 | 4.695329 | 1.21526 | 8.163844 | 0.012547 |
| left inferior frontal superior frontal cortex | -8.72E-05 | -0.00012 | -5.90E-05 | 2.98E-08 | 6.315462 | 2.988792 | 9.631788 | 0.001161 |
| right fornix, excluding fimbria | -0.00021 | -0.00035 | -6.74E-05 | 0.003987 | 1.376882 | -0.39231 | 3.132066 | 0.125593 |
| left fornix, excluding fimbria | -0.00044 | -0.00062 | -0.00026 | 2.61E-06 | 2.14186 | 0.31036 | 3.961593 | 0.024353 |
| all DTI atlas tract fibers | -5.64E-06 | -7.61E-06 | -3.67E-06 | 1.35E-07 | 4.875087 | 1.915424 | 7.821476 | 0.003633 |
| right hemisphere DTI atlas tract fibers without corpus callosum | -1.72E-05 | -2.28E-05 | -1.16E-05 | 2.98E-08 | 5.603851 | 2.347912 | 8.846244 | 0.002758 |
| left hemisphere DTI atlas tract fibers without corpus callosum | -1.64E-05 | -2.20E-05 | -1.08E-05 | 1.06E-07 | 6.367129 | 3.055527 | 9.666148 | 0.001114 |
| right hemisphere DTI atlas tract fibers | -1.12E-05 | -1.50E-05 | -7.28E-06 | 1.35E-07 | 4.526513 | 1.612178 | 7.42713 | 0.005026 |

|  | Radial diffusivity | | | | Neurite density | | | |
| --- | --- | --- | --- | --- | --- | --- | --- | --- |
| dict | Coefficient | 95% CI | | Adjusted p-value | Coefficient | 95% CI | | Adjusted p-value |
| right fornix | 2.575754 | 0.474302 | 4.664731 | 0.01811 | -0.90748 | -2.71208 | 0.903117 | 0.381583 |
| left fornix | 2.446024 | 0.330326 | 4.550668 | 0.025494 | -1.1649 | -2.97688 | 0.653044 | 0.274386 |
| right cingulate cingulum | 2.291788 | 0.438378 | 4.140979 | 0.017777 | -0.93783 | -2.14223 | 0.269809 | 0.181077 |
| left cingulate cingulum | 2.265367 | 0.254096 | 4.270158 | 0.029074 | -1.03328 | -2.35808 | 0.297295 | 0.181077 |
| right parahippocampal cingulum | 2.275663 | 0.510659 | 4.031939 | 0.013939 | -1.6438 | -3.02153 | -0.26269 | 0.033952 |
| left parahippocampal cingulum | 2.321893 | 0.547635 | 4.086575 | 0.012857 | -1.62582 | -2.9746 | -0.27222 | 0.033002 |
| right corticospinal/pyramidal | 6.196824 | 3.077502 | 9.317692 | 0.000294 | -4.66531 | -7.38958 | -1.99733 | 0.003366 |
| left corticospinal/pyramidal | 6.489113 | 3.433888 | 9.542872 | 0.00013 | -4.66415 | -7.35364 | -2.02709 | 0.003182 |
| right anterior thalamic radiations | 4.990235 | 1.936183 | 8.034139 | 0.00204 | -1.6212 | -3.68095 | 0.442697 | 0.181077 |
| left anterior thalamic radiations | 4.930743 | 1.930354 | 7.919893 | 0.002016 | -1.53296 | -3.57084 | 0.510094 | 0.194981 |
| right uncinate | 4.014652 | 1.814148 | 6.206485 | 0.000678 | -2.5662 | -4.2656 | -0.86373 | 0.008816 |
| left uncinate | 2.815041 | 0.539163 | 5.080253 | 0.017777 | -1.38871 | -3.01965 | 0.246862 | 0.147436 |
| right inferior longitudinal fasiculus | 3.484493 | 0.896415 | 6.066501 | 0.010806 | -2.04321 | -3.88045 | -0.20191 | 0.04967 |
| left inferior longitudinal fasiculus | 4.244191 | 1.69161 | 6.791077 | 0.001928 | -2.87864 | -4.68264 | -1.0709 | 0.006399 |
| right inferior-fronto-occipital fasiculus | 4.142125 | 1.627694 | 6.646255 | 0.002016 | -2.65054 | -4.52411 | -0.77085 | 0.013814 |
| left inferior-fronto-occipital fasiculus | 4.405806 | 1.839304 | 6.964094 | 0.00137 | -2.88177 | -4.7757 | -0.98321 | 0.008672 |
| foreceps major | 1.584249 | -0.22888 | 3.383799 | 0.087635 | -0.81572 | -2.32318 | 0.724195 | 0.358332 |
| foreceps minor | 3.003206 | 1.158407 | 4.834765 | 0.00204 | -2.34942 | -3.87052 | -0.81758 | 0.008097 |
| corpus callosum | 3.763262 | 1.292181 | 6.222676 | 0.003748 | -2.88477 | -5.11767 | -0.64284 | 0.023853 |
| right superior longitudinal fasiculus | 5.092627 | 2.145145 | 8.027685 | 0.001312 | -2.91063 | -5.02622 | -0.78522 | 0.016023 |
| left superior longitudinal fasiculus | 5.398619 | 2.559399 | 8.226656 | 0.000438 | -3.19488 | -5.2285 | -1.15138 | 0.007157 |
| right temporal superior longitudinal fasiculus | 3.141264 | 0.267026 | 6.001325 | 0.033276 | -1.22922 | -3.30007 | 0.855605 | 0.316307 |
| left temporal superior longitudinal fasiculus | 4.460979 | 1.662832 | 7.246663 | 0.002509 | -2.47303 | -4.49488 | -0.43871 | 0.031292 |
| right parietal superior longitudinal fasiculus | 5.774376 | 2.83771 | 8.69939 | 0.000309 | -3.43168 | -5.5199 | -1.33452 | 0.005421 |
| left parietal superior longitudinal fasiculus | 6.621251 | 3.787235 | 9.445872 | 3.13E-05 | -3.935 | -5.88819 | -1.97538 | 0.000836 |
| right superior corticostriate | 7.786399 | 4.310456 | 11.26555 | 6.64E-05 | -3.02662 | -5.05752 | -0.99806 | 0.009353 |
| left superior corticostriate | 10.69457 | 6.928754 | 14.4637 | 1.10E-06 | -5.35704 | -7.75945 | -2.95985 | 0.000326 |
| right superior corticostriate-frontal cortex only | 7.110841 | 3.493615 | 10.73601 | 0.000309 | -2.56955 | -4.64604 | -0.49677 | 0.029189 |
| left superior corticostriate-frontal cortex only | 10.02266 | 6.319041 | 13.72627 | 2.36E-06 | -4.69509 | -6.9808 | -2.41282 | 0.000752 |
| right superior corticostriate-parietal cortex only | 6.896922 | 3.632911 | 10.16245 | 0.000132 | -2.92287 | -4.90002 | -0.94647 | 0.009635 |
| left superior corticostriate-parietal cortex only | 8.745114 | 5.379119 | 12.11377 | 4.97E-06 | -4.55014 | -6.72798 | -2.37615 | 0.000744 |
| right striatal inferior frontal cortex | 4.28001 | 1.937647 | 6.610977 | 0.000678 | -2.19232 | -3.87527 | -0.50472 | 0.023263 |
| left striatal inferior frontal cortex | 4.699818 | 2.204028 | 7.183885 | 0.000476 | -2.5391 | -4.34618 | -0.72598 | 0.014001 |
| right inferior frontal superior frontal cortex | 6.454758 | 3.217829 | 9.683894 | 0.000293 | -3.58417 | -5.82256 | -1.34149 | 0.006399 |
| left inferior frontal superior frontal cortex | 7.253329 | 4.192312 | 10.30664 | 2.77E-05 | -4.18008 | -6.29851 | -2.05676 | 0.000861 |
| right fornix, excluding fimbria | 1.302677 | -0.75373 | 3.350335 | 0.213344 | 0.466396 | -1.06781 | 2.002509 | 0.568058 |
| left fornix, excluding fimbria | 3.11414 | 1.080656 | 5.140266 | 0.003688 | -1.65202 | -3.28169 | -0.0213 | 0.076874 |
| all DTI atlas tract fibers | 6.529734 | 3.34493 | 9.703288 | 0.000198 | -5.3287 | -8.17627 | -2.47547 | 0.001484 |
| right hemisphere DTI atlas tract fibers without corpus callosum | 7.764014 | 4.273169 | 11.24515 | 6.64E-05 | -5.61363 | -8.53435 | -2.6893 | 0.001122 |
| left hemisphere DTI atlas tract fibers without corpus callosum | 8.790745 | 5.252936 | 12.32028 | 1.13E-05 | -6.53846 | -9.43253 | -3.64245 | 0.000326 |
| right hemisphere DTI atlas tract fibers | 6.039335 | 2.913913 | 9.15305 | 0.000364 | -4.7608 | -7.56939 | -1.94572 | 0.004062 |
| left hemisphere DTI atlas tract fibers | 6.793744 | 3.617615 | 9.959045 | 0.000124 | -5.59814 | -8.40367 | -2.78756 | 0.000836 |
| left thalamus proper |  |  |  |  | 0.012365 | -2.70617 | -4.83109 | 0.667687 |
| left caudate |  |  |  |  | 0.539367 | 0.42063 | -0.93122 | 1 |
| left putamen |  |  |  |  | 0.29861 | -0.94043 | -2.7245 | 1 |
| left pallidum |  |  |  |  | 0.197772 | 0.56208 | -0.29685 | 1 |
| left hippocampus |  |  |  |  | 0.513402 | -0.75699 | -3.03918 | 1 |
| left amygdala |  |  |  |  | 0.367631 | -0.95205 | -3.03197 | 1 |
| right thalamus proper |  |  |  |  | 0.086099 | -2.349 | -5.033 | 1 |
| right caudate |  |  |  |  | 0.273386 | 0.764055 | -0.61236 | 1 |
| right putamen |  |  |  |  | 0.557538 | 0.458643 | -1.08431 | 1 |
| right pallidum |  |  |  |  | 0.57515 | 0.216936 | -0.54605 | 1 |
| right hippocampus |  |  |  |  | 0.49018 | -0.82945 | -3.19813 | 1 |
| right amygdala |  |  |  |  | 0.514202 | -0.70283 | -2.82351 | 1 |

**3.3. rest-state fMRI**

| dict | Coefficient | 95% CI | | Adjusted p-value |
| --- | --- | --- | --- | --- |
| auditory network and auditory network | -0.66198 | -1.2188 | -0.11043 | 0.067213 |
| auditory network and cingulo-opercular network | 0.024244 | -0.70931 | 0.750985 | 0.969405 |
| auditory network and cingulo-parietal network | 0.720204 | 0.20776 | 1.231848 | 0.02697 |
| auditory network and default network | 0.135823 | -0.71212 | 0.986894 | 0.851008 |
| auditory network and dorsal attention network | 0.167181 | -0.72666 | 1.060826 | 0.822294 |
| auditory network and fronto-parietal network | 0.04437 | -0.83134 | 0.922199 | 0.952372 |
| auditory network and "none" network | 0.815426 | -0.61 | 2.248106 | 0.42061 |
| auditory network and retrosplenial temporal network | 0.778832 | 0.19446 | 1.362775 | 0.037063 |
| auditory network and sensorimotor hand network | -1.30549 | -1.99404 | -0.62227 | 0.002169 |
| auditory network and sensorimotor mouth network | -0.7466 | -1.26595 | -0.23145 | 0.024782 |
| auditory network and salience network | 0.561393 | 0.025882 | 1.096233 | 0.120582 |
| auditory network and ventral attention network | 1.045217 | 0.27424 | 1.815786 | 0.034011 |
| auditory network and visual network | 0.191552 | -0.45518 | 0.837515 | 0.785782 |
| cingulo-opercular network and cingulo-opercular network | -0.92704 | -1.57629 | -0.28453 | 0.024782 |
| cingulo-opercular network and cingulo-parietal network | -0.61636 | -1.17055 | -0.06435 | 0.097502 |
| cingulo-opercular network and default network | 1.802395 | 1.009077 | 2.598403 | 0.000398 |
| cingulo-opercular network and dorsal attention network | -0.7646 | -1.68629 | 0.147868 | 0.241016 |
| cingulo-opercular network and fronto-parietal network | 0.334879 | -0.56417 | 1.234944 | 0.688773 |
| cingulo-opercular network and "none" network | 3.176249 | 1.709497 | 4.65314 | 0.000532 |
| cingulo-opercular network and retrosplenial temporal network | 0.954066 | 0.353008 | 1.556184 | 0.013146 |
| cingulo-opercular network and sensorimotor hand network | -0.0545 | -0.88921 | 0.776305 | 0.952372 |
| cingulo-opercular network and sensorimotor mouth network | 0.060356 | -0.51884 | 0.636372 | 0.907501 |
| cingulo-opercular network and salience network | 0.147984 | -0.43854 | 0.732855 | 0.801954 |
| cingulo-opercular network and ventral attention network | 1.49811 | 0.692389 | 2.304939 | 0.002727 |
| cingulo-opercular network and visual network | -0.64807 | -1.33725 | 0.040374 | 0.179619 |
| cingulo-parietal network and cingulo-parietal network | -0.28279 | -0.50553 | -0.06042 | 0.048339 |
| cingulo-parietal network and default network | 0.005414 | -0.55199 | 0.562882 | 0.98481 |
| cingulo-parietal network and dorsal attention network | -0.07994 | -0.62986 | 0.470373 | 0.860869 |
| cingulo-parietal network and fronto-parietal network | -0.34235 | -0.94184 | 0.258484 | 0.42061 |
| cingulo-parietal network and "none" network | 0.637689 | -0.35581 | 1.634898 | 0.38396 |
| cingulo-parietal network and retrosplenial temporal network | 0.053496 | -0.33475 | 0.441081 | 0.862745 |
| cingulo-parietal network and sensorimotor hand network | 0.552108 | 0.01562 | 1.089202 | 0.128486 |
| cingulo-parietal network and sensorimotor mouth network | 0.23446 | -0.14124 | 0.609623 | 0.393996 |
| cingulo-parietal network and salience network | -0.57116 | -0.94676 | -0.1958 | 0.018609 |
| cingulo-parietal network and ventral attention network | 0.204774 | -0.37863 | 0.789718 | 0.710548 |
| cingulo-parietal network and visual network | 0.329555 | -0.10719 | 0.765048 | 0.296358 |
| default network and default network | -1.08737 | -1.81999 | -0.36109 | 0.01978 |
| default network and dorsal attention network | 2.299711 | 1.480249 | 3.123402 | 3.72E-06 |
| default network and fronto-parietal network | 1.52374 | 0.588333 | 2.455219 | 0.011354 |
| default network and "none" network | -1.78518 | -3.21019 | -0.37255 | 0.049686 |
| default network and retrosplenial temporal network | -0.54085 | -1.20327 | 0.118215 | 0.241016 |
| default network and sensorimotor hand network | 0.627485 | -0.28661 | 1.54385 | 0.346508 |
| default network and sensorimotor mouth network | -0.15946 | -0.79488 | 0.480384 | 0.801954 |
| default network and salience network | 0.164499 | -0.46623 | 0.792692 | 0.801954 |
| default network and ventral attention network | -1.18899 | -2.03993 | -0.34599 | 0.02697 |
| default network and visual network | 0.278158 | -0.47198 | 1.035059 | 0.688773 |
| dorsal attention network and dorsal attention network | -1.32985 | -2.02071 | -0.64485 | 0.002169 |
| dorsal attention network and fronto-parietal network | -0.82738 | -1.81403 | 0.155386 | 0.241016 |
| dorsal attention network and "none" network | 3.145925 | 1.510971 | 4.792596 | 0.002169 |
| dorsal attention network and retrosplenial temporal network | 0.389384 | -0.28948 | 1.068924 | 0.42061 |
| dorsal attention network and sensorimotor hand network | 0.817614 | -0.10781 | 1.738975 | 0.221021 |
| dorsal attention network and sensorimotor mouth network | 0.413113 | -0.23474 | 1.060241 | 0.38396 |
| dorsal attention network and salience network | -0.33491 | -0.96847 | 0.297002 | 0.469833 |
| dorsal attention network and ventral attention network | 1.882268 | 1.013512 | 2.75651 | 0.000532 |
| dorsal attention network and visual network | -0.48254 | -1.2283 | 0.259809 | 0.38396 |
| fronto-parietal network and fronto-parietal network | -0.79955 | -1.55685 | -0.04916 | 0.118007 |
| fronto-parietal network and "none" network | 0.084997 | -1.58156 | 1.7433 | 0.952372 |
| fronto-parietal network and retrosplenial temporal network | 0.597641 | -0.11835 | 1.315786 | 0.241016 |
| fronto-parietal network and sensorimotor hand network | 1.193813 | 0.261642 | 2.129159 | 0.048285 |
| fronto-parietal network and sensorimotor mouth network | 0.177999 | -0.50012 | 0.858723 | 0.801954 |
| fronto-parietal network and salience network | -0.47875 | -1.11608 | 0.15588 | 0.296358 |
| fronto-parietal network and ventral attention network | 1.509347 | 0.591692 | 2.425943 | 0.011354 |
| fronto-parietal network and visual network | 0.160283 | -0.64848 | 0.974035 | 0.816662 |
| "none" network and "none" network | -1.02452 | -2.75078 | 0.671555 | 0.412872 |
| "none" network and retrosplenial temporal network | -0.53521 | -1.62893 | 0.551611 | 0.518024 |
| "none" network and sensorimotor hand network | 0.358959 | -1.14404 | 1.870711 | 0.8075 |
| "none" network and sensorimotor mouth network | 0.206317 | -0.83897 | 1.260091 | 0.816662 |
| "none" network and salience network | 0.580059 | -0.4309 | 1.587313 | 0.42061 |
| "none" network and ventral attention network | -2.49898 | -4.08295 | -0.93442 | 0.013146 |
| "none" network and visual network | 0.065765 | -1.13769 | 1.276559 | 0.952372 |
| retrosplenial temporal network and retrosplenial temporal network | -0.6626 | -1.00925 | -0.31719 | 0.002169 |
| retrosplenial temporal network and sensorimotor hand network | -0.31835 | -0.99049 | 0.353794 | 0.535617 |
| retrosplenial temporal network and sensorimotor mouth network | -0.0909 | -0.55301 | 0.3719 | 0.816662 |
| retrosplenial temporal network and salience network | 0.321284 | -0.11509 | 0.757509 | 0.3013 |
| retrosplenial temporal network and ventral attention network | 0.514723 | -0.10917 | 1.136483 | 0.241016 |
| retrosplenial temporal network and visual network | -0.12393 | -0.62254 | 0.373565 | 0.801954 |
| sensorimotor hand network and sensorimotor hand network | -0.49317 | -1.0613 | 0.068599 | 0.226284 |
| sensorimotor hand network and sensorimotor mouth network | -0.46992 | -0.94931 | 0.00662 | 0.153395 |
| sensorimotor hand network and salience network | 0.416867 | -0.18373 | 1.017949 | 0.34382 |
| sensorimotor hand network and ventral attention network | 0.192906 | -0.64837 | 1.037641 | 0.8075 |
| sensorimotor hand network and visual network | -0.4184 | -1.10145 | 0.265235 | 0.402577 |
| sensorimotor mouth network and sensorimotor mouth network | -0.31012 | -0.59762 | -0.02487 | 0.10974 |
| sensorimotor mouth network and salience network | 0.108369 | -0.31885 | 0.535953 | 0.801954 |
| sensorimotor mouth network and ventral attention network | 0.128496 | -0.50058 | 0.760905 | 0.816662 |
| sensorimotor mouth network and visual network | -0.01067 | -0.51246 | 0.488542 | 0.977416 |
| salience network and salience network | -0.22421 | -0.52979 | 0.079353 | 0.3013 |
| salience network and ventral attention network | 0.483665 | -0.10522 | 1.070218 | 0.241016 |
| salience network and visual network | 0.083 | -0.4432 | 0.610674 | 0.851008 |
| ventral attention network and ventral attention network | -1.02217 | -1.70369 | -0.34793 | 0.018914 |
| ventral attention network and visual network | 0.166986 | -0.5673 | 0.905428 | 0.8075 |
| visual network and visual network | -0.13757 | -0.57728 | 0.29905 | 0.765322 |

**4. References**

Avery, E.W., Behland, J., Mak, A., Haider, S.P., Zeevi, T., Sanelli, P.C., et al. (2022). CT angiographic radiomics signature for risk stratification in anterior large vessel occlusion stroke. *Neuroimage Clin* 34**,** 103034. doi: 10.1016/j.nicl.2022.103034.

Breiman, L. (2001). Random Forests. *Machine Learning* 45(1)**,** 5-32. doi: 10.1023/A:1010933404324.

David Meyer, E.D., Kurt Hornik, Andreas Weingessel and Friedrich Leisch (2022). e1071: Misc Functions of the Department of Statistics, Probability

Theory Group (Formerly: E1071),. [*https://CRAN.R-project.org/package=e1071*](https://CRAN.R-project.org/package=e1071) R package version 1.7-11.

De Jay, N., Papillon-Cavanagh, S., Olsen, C., El-Hachem, N., Bontempi, G., and Haibe-Kains, B. (2013). mRMRe: an R package for parallelized mRMR ensemble feature selection. *Bioinformatics* 29(18)**,** 2365-2368. doi: 10.1093/bioinformatics/btt383.

Friedman, J., Hastie, T., and Tibshirani, R. (2010). Regularization Paths for Generalized Linear Models via Coordinate Descent. *J Stat Softw* 33(1)**,** 1-22.

Haider, S.P., Mahajan, A., Zeevi, T., Baumeister, P., Reichel, C., Sharaf, K., et al. (2020). PET/CT radiomics signature of human papilloma virus association in oropharyngeal squamous cell carcinoma. *Eur J Nucl Med Mol Imaging* 47(13)**,** 2978-2991. doi: 10.1007/s00259-020-04839-2.

Kuhn, M. (2022). caret: Classification and Regression Training. *R package version 6.0-93* <https://CRAN.R-project.org/package=caret>.

Majka, M. (2019). naivebayes: High Performance Implementation of the Naive Bayes Algorithm in R. R package version 0.9.7. [*https://CRAN.R-project.org/package=naivebayes*](https://CRAN.R-project.org/package=naivebayes).

Murtagh, F., and Legendre, P. (2014). Ward’s Hierarchical Agglomerative Clustering Method: Which Algorithms Implement Ward’s Criterion? *Journal of Classification* 31(3)**,** 274-295. doi: 10.1007/s00357-014-9161-z.

R Development Core Team (2019). "R: A language and environment for statistical computing". (Vienna, Austria: R Foundation for Statistical Computing).

Tianqi Chen, T.H., Michael Benesty, Vadim Khotilovich, Yuan Tang, Hyunsu Cho, Kailong Chen, Rory Mitchell , Ignacio Cano, Tianyi Zhou, Mu Li, Junyuan Xie, Min Lin, Yifeng Geng, Yutian Li, Jiaming Yuan (2022). Package 'xgboost'. *ed. CRAN*.

Tianqi Chen, T.H., Michael Benesty, Vadim Khotilovich, Yuan Tang, Hyunsu Cho, Kailong Chen, Rory Mitchell, Ignacio Cano, Tianyi Zhou, Mu Li, Junyuan Xie, Min Lin, Yifeng Geng and Yutian Li (2021). xgboost: Extreme Gradient Boosting.

Weber, C.F., Lake, E.M.R., Haider, S.P., Mozayan, A., Mukherjee, P., Scheinost, D., et al. (2022). Age-dependent white matter microstructural disintegrity in autism spectrum disorder. *Front Neurosci* 16**,** 957018. doi: 10.3389/fnins.2022.957018.
